# Genome-wide Association Study of Idiopathic Osteonecrosis of the Femoral Head

**DOI:** 10.1038/s41598-017-14778-y

**Published:** 2017-11-08

**Authors:** Yuma Sakamoto, Takuaki Yamamoto, Nobuhiko Sugano, Daisuke Takahashi, Toshiyuki Watanabe, Takashi Atsumi, Junichi Nakamura, Yukiharu Hasegawa, Koichi Akashi, Ichiei Narita, Takeshi Miyamoto, Tsutomu Takeuchi, Katsunori Ikari, Koichi Amano, Atsuhiro Fujie, Toshikazu Kubo, Yoshifumi Tada, Ayumi Kaneuji, Hiroaki Nakamura, Tomoya Miyamura, Tamon Kabata, Ken Yamaji, Takahiro Okawa, Akihiro Sudo, Kenji Ohzono, Yoshiya Tanaka, Yuji Yasunaga, Shuichi Matsuda, Yuuki Imai, Japanese Research Committee on Idiopathic Osteonecrosis of the Femoral Head, Yasuharu Nakashima, Goro Motomura, Satoshi Ikemura, Ryosuke Yamaguchi, Kazuyuki Karasuyama, Kazuhiko Sonoda, Takashi Nishii, Takashi Sakai, Masaki Takao, Tohru Irie, Tsuyoshi Asano, Norimasa Iwasaki, Tatsuya Atsumi, Satoshi Tamaoki, Ryosuke Nakanishi, Satoe Tanabe, Shunji Kishida, Shigeo Hagiwara, Taisuke Seki, Hiroshi Tsukamoto, Hiroaki Niiro, Yojiro Arinobu, Mitsuteru Akahoshi, Hiroshi Mitoma, Masahiro Ayano, Takeshi Kuroda, Yoshiaki Toyama, Atsushi Funayama, Hironari Hanaoka, Kunihiro Yamaoka, Yasushi Kawaguchi, Hisashi Yamanaka, Tetsuji Hosozawa, Shigeki Momohara, Kentaro Chino, Mikihiro Fujioka, Keichiro Ueshima, Masashi Ishida, Masazumi Saito, Shigeki Hayashi, Akira Ikegami, Toru Ichiseki, Shigekazu Mizokawa, Yoichi Ohta, Yoshitomo Kajino, Fumio Sekiya, Fujio Higuchi, Masahiro Hasegawa, Noriki Miyamoto, Shinichi Miyazaki, Toshio Yamaguchi, Wataru Ando, Kazuyoshi Saito, Kazuhisa Nakano, Yutaka Kuroda, Takuma Yamasaki, Masato Akiyama, Michiaki Kubo, Yoichiro Kamatani, Yukihide Iwamoto, Shiro Ikegawa

**Affiliations:** 1 Department of Orthopaedic Surgery, Graduate School of Medical Sciences, Kyushu University, 3-1-1 Maidashi, Higashi-ku, Fukuoka-city, Fukuoka 812-8582 Japan; 4 Department of Orthopaedic Medical Engineering, Osaka University Graduate School of Medicine, 2-2 Yamadaoka, Suita-city, Osaka 565-0871 Japan; 5 Department of Orthopaedic Surgery, Hokkaido University Graduate School of Medicine, Kita 15, Nishi 7, Kita-ku, Sapporo-city, Hokkaido 060-8638 Japan; 6 Division of Rheumatology, Endocrinology and Nephrology, Hokkaido University Graduate School of Medicine, Kita 15, Nishi 7, Kita-ku, Sapporo-city, Hokkaido 060-8638 Japan; 7 Department of Orthopaedic Surgery, Fujigaoka Hospital, Showa University School of Medicine, 1-30 Fujigaoka, Aoba-ku, Yokohama-city, Kanagawa 227-8501 Japan; 8 Department of Orthopaedic Surgery, Graduate School of Medicine, Chiba University, 1-8-1 Inohana, Chuo-ku, Chiba-city, Chiba 260-8677 Japan; 9 Department of Orthopaedic Surgery, Nagoya University Graduate School of Medicine, 65 Tsurumai-cho, Showa-ku, Nagoya-city, Aichi 466-8550 Japan; 10 Department of Medicine and Biosystemic Science, Kyushu University Graduate School of Medical Sciences, 3-1-1 Maidashi, Higashi-ku, Fukuoka-city, Fukuoka 812-8582 Japan; 11 Division of Clinical Nephrology and Rheumatology, Kidney Research Center, Niigata University Graduate School of Medical and Dental Sciences, 1-757 Chuo-ku, Niigata-city, Niigata 951-8510 Japan; 12 Department of Orthopedic Surgery, Keio University School of Medicine, 35 Shinano-machi, Shinjuku-ku, Tokyo 160-8582 Japan; 13 Division of Rheumarology, Department of Internal Medicine, Keio University School of Medicine, 35 Shinano-machi, Shinjuku-ku, Tokyo 160-8582 Japan; 14 Institute of Rheumatology, Tokyo Women’s Medical University, 10-22 Kawada-cho, Shinjuku-ku, Tokyo 162-0054 Japan; 15 Department of Rheumatology and Clinical Immunology, Saitama Medical Center, Saitama Medical University, 1981 Kamoda, Kawagoe-city, Saitama 350-8550 Japan; 16 Department of Orthopaedics, Graduate School of Medical Science, Kyoto Prefectural University of Medicine, 465 Kajii-cho, Kawaramachi-Hirokoji, Kamigyo-ku, Kyoto-city, Kyoto 602-8566 Japan; 18 Department of Orthopaedic Surgery, Kanazawa Medical University, 1-1 Daigaku, Uchinada-machi, Kahoku-gun, Ishikawa 920-0293 Japan; 19 Department of Orthopaedic Surgery, Osaka City University Graduate School of Medicine, 1-4-3, Asahi-machi, Abeno-ku, Osaka-city, Osaka 545-8585 Japan; 21 Department of Orthopaedic Surgery, Graduate School of Medical Science, Kanazawa University, Takara-machi 13-1, Kanazawa-city, Ishikawa 920-8641 Japan; 22 Department of Internal Medicine and Rheumatology, Juntendo University School of Medicine, 2-1-1 Hongo, Bunkyo-ku, Tokyo 113-8421 Japan; 23 Department of Orthopaedic Surgery, Kurume University Medical Center, 155-1 Kokubu-machi, Kurume-city, Fukuoka 839-0863 Japan; 24 Department of Orthopaedic Surgery, Mie University Graduate School of Medicine, 2-174 Edobashi, Tsu-city, Mie 514-8507 Japan; 25 Department of Orthopaedic Surgery, Kansai Rosai Hospital, 1-69 Inabasou 3-chome, Amagasaki-city, Hyogo 660-8511 Japan; 26 The First Department of Internal Medicine, University of Occupational and Environmental Health, School of Medicine, 1-1 Iseigaoka, Yahatanishi-ku, Kitakyushu-city, Fukuoka 807-8555 Japan; 28 Department of Orthopaedic Surgery, Graduate School of Medicine, Kyoto University, 54 Kawahara-cho, Shogoin, Sakyo-ku, Kyoto-city, Kyoto 606-8507 Japan; 33 Department of Artificial Joints and Biomaterials, Institute of Biomedical and Health Sciences, Hiroshima University, Hiroshima-city, Hiroshima Japan; 2 Laboratory for Bone and Joint Diseases, RIKEN Center for Integrative Medical Sciences, 4-6-1 Shirokanedai, Minato-ku, Tokyo 108-8639 Japan; 3 Department of Orthopaedic Surgery, Faculty of Medicine, Fukuoka University, 7-45-1 Nanakuma, Jonan-ku, Fukuoka-city, Fukuoka 814-0180 Japan; 17 Department of Rheumatology, Faculty of Medicine, Saga University, 5-1-1 Nabeshima, Saga-city, Saga 849-8501 Japan; 20 Department of Internal Medicine and Rheumatology, National Hospital Organization Kyushu Medical Center, 1-8-1 Jigyohama, Chuo-ku, Fukuoka-city, Fukuoka 810-8563 Japan; 27 Department of Orthopaedic Surgery, Hiroshima Prefectural Rehabilitation Center, Taguchi 295-3, Saijo-cho, Higashi-Hiroshima-city, Hiroshima 739-0036 Japan; 29 Division of Integrative Pathophysiology, Proteo-Science Center, Ehime University Graduate School of Medicine, Shitsukawa, Toon-city, Ehime 791-0295 Japan; 30 Laboratory for Statistical Analysis, RIKEN Center for Integrative Medical Sciences, 1-7-22 Suehiro-cho, Tsurumi-ku, Yokohama-city, Kanagawa 230-0045 Japan; 31 RIKEN Center for Integrative Medical Sciences, 1-7-22 Suehiro-cho, Tsurumi-ku, Yokohama-city, Kanagawa 230-0045 Japan; 32 Department of Orthopaedic Surgery, Kyushu Rosai Hospital, 1-1 Sonekita-machi, Kokuraminami-ku, Kitakyushu-city, Fukuoka 800-0229 Japan

## Abstract

Idiopathic osteonecrosis of the femoral head (IONFH) is an ischemic disorder that causes bone necrosis of the femoral head, resulting in hip joint dysfunction. IONFH is a polygenic disease and steroid and alcohol have already known to increase its risk; however, the mechanism of IONFH remains to be elucidated. We performed a genome-wide association study using ~60,000 subjects and found two novel loci on chromosome 20q12 and 12q24. Big data analyses identified *LINC01370* as a candidate susceptibility gene in the 20q12 locus. Stratified analysis by IONFH risk factors suggested that the 12q24 locus was associated with IONFH through drinking capacity. Our findings would shed new light on pathophysiology of IONFH.

## Introduction

Osteonecrosis of the femoral head (ONFH) is an ischemic disorder that presents with bone necrosis of the femoral head^1^. ONFH often leads to a collapse of the femoral head, which results in osteoarthritis and eventually requires surgical treatment^2^. ONFH with evident cause of ischemia, such as trauma, radiation, decompression sickness, and sickle cell disease is classified as secondary ONFH. On the other hand, ONFH without evident cause is referred to as idiopathic ONFH (IONFH). IONFH is a relatively common disease throughout the world, and the number of IONFH patients is increasing year by year. Its estimated number during 2004 is 11,400 (ref.^3^) and the annual incidence rate is 2.51 cases per 100,000 person-years in Japan^4^. In the United States, ONFH is estimated to develop in 10,000 to 20,000 patients in a year^1,2^.

The pathophysiology of IONFH has not been elucidated. Epidemiological studies have clearly indicated the two major risk factors: massive steroid treatment and heavy alcohol drinking^2,3^. Thus, IONFH is further classified into three groups; ‘Steroid-associated’, ‘Alcohol-associated’ and ‘Neither steroid nor alcohol-associated (Neither-associated)’ ONFH^5^, the proportion of which in Japan is about 51%, 31% and 15%, respectively^3^. The most frequent underlying disease for steroid-associated ONFH is systemic lupus erythematosus (SLE).

Some people without steroid treatment or alcohol drinking can develop IONFH, namely, neither-associated ONFH. On the other hand, many people with steroid treatment or alcohol drinking do not develop ONFH. For example, 66.7–68.1% of SLE patients with steroid treatment do not develop steroid-associated ONFH^6,7^; 94.6–99.7% of heavy alcohol-drinkers do not develop alcohol-associated ONFH^8,9^. These data suggest individual differences in the susceptibility for IONFH. One explanation of such differences in the susceptibility is the genetic factor. Identical twins are reported in steroid-associated and neither-associated ONFHs^10,11^. A national survey in China reported that a family history of ONFH is positively associated with an increased risk of non-traumatic ONFH^12^. In addition, it has been reported that ONFH is caused by *COL2A1* mutations^13^. These findings suggest that genetic factors can influence the susceptibility of IONFH.

In order to identify the genetic factor of IONFH, many candidate gene analyses have been performed^14^; however, these studies have only examined specific SNPs in candidate genes related to plausible pathogenesis of ONFH, such as coagulation^15^, fibrinolysis^16^, angiogenesis^17^, hypoxic response^18^, and steroid^19^ and alcohol metabolisms^20^. No definite susceptibility genes have been identified yet. There have been two genome-wide association study (GWAS) reports from a group for steroid-associated ONFH, which reported significant loci around *GRIN3A* (glutamate ionotropic receptor NMDA type subunit 3 A)^21^, *BMP7* (bone morphogenic protein 7), *LINC00251* (long intergenic non-protein coding RNA 251) and *PROX1-AS1* (PROX1 antisense RNA 1)^22^. However, the study was limited mostly to children with acute lymphoblastic leukemia while IONFH commonly occurs in 30’s to 50’s (ref.^3^), and the sample size of the study was small (the number of the case: 400 and 102) to obtain a definitive conclusion. The genetic landscape of ION remains unclear and a large-scale GWAS is awaited.

In this study, we performed a GWAS for IONFH in Japanese using 1,602 cases and 60,000 controls. We identified a 20q12 locus with genome-wide significance and found *LINC01370* (long intergenic non-protein coding RNA 1370) as a candidate susceptibility gene for IONFH. We also identified a 12q24 locus that might be associated with IONFH through its association with drinking capacity. Stratified analysis by the group showed different patterns of association plots in each group, which probably reflected the underlying difference in their pathophysiology as well as the risk factors of IONFH.

## Results

### GWAS and imputation

IONFH cases were diagnosed based on the criteria of Japanese Research Committee (JRC) on IONFH in the Ministry of Health, Labour and Welfare of Japan. We collected DNA samples from 1,602 IONFH cases with clinical information. As controls, we used 60,000 genome-wide screening data in the BioBank Japan Project^23^. After the sample quality control (QC), 1,547 cases and 59,103 controls remained (Table S1 and Fig. S1). Over 520,000 autosomal SNPs passed the standard QC of genotyped SNP (Table 1).

**Table 1:** Numbers of GWAS samples and SNPs after quality control.

Phenotype	Case			Control^1^			Genotyped SNP
	Sample	Male	Female	Sample	Male	Female	
IONFH	1,547	777	770	59,103	32,135	26,968	525,308
***Stratified analysis based on risk factors*** ^2^							
Steroid-associated	1,058	386	672	59,103	32,135	26,968	525,264
Alcohol-associated	351	316	35	59,103	32,135	26,968	524,775
Neither-associated	132	70	62	59,103	32,135	26,968	523,825
***Stratified analysis based on alcohol intake*** ^3^							
Alcohol-associated	351	316	35	3,647	3,349	298	525,254

^1^Derived from BioBank Japan. ^2^Six patients could not be classified because sufficient information for classification could not be obtained. ^3^Controls were matched for alcohol-drinking history (400 ml or more ethanol consumption per week). IONFH: idiopathic osteonecrosis of the femoral head.

**Figure 1:**
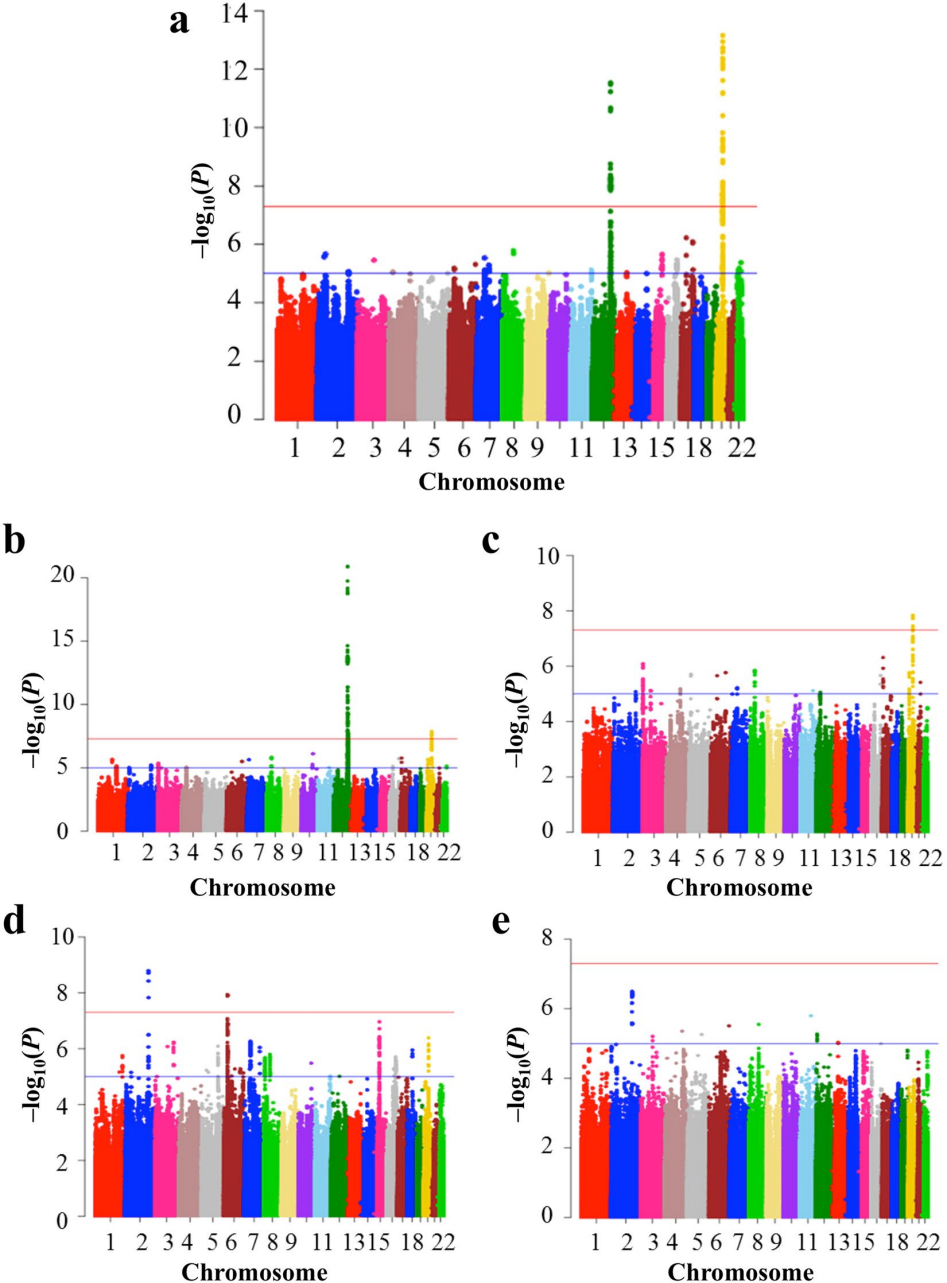
Manhattan plot of imputed data (**a**) Idiopathic osteonecrosis of the femoral head vs BioBank Japan (BBJ), (**b**) alcohol-associated osteonecrosis of the femoral head (ONFH) vs BBJ, (**c**) alcohol-associated ONFH vs BBJ of heavy drinker (400 ml/day or more ethanol consumption), (**d**) steroid-associated ONFH vs BBJ, and (**e**) neither-associated ONFH vs BBJ. The SNPs with genome-wide significance were identified in (**a**) 12q24.11–12 and 20q12, (**b**) 12q24.11–13 and 20q12, (**c**) 20q12, and (**d**) 2q32 and 6p21. The red and blue lines represented the threshold of genome-wide significance (*P* = 5 × 10^−8^) and suggestive association threshold (*P* = 1 × 10^−5^), respectively.

We performed GWAS for IONFH using gender and top 10 principal component scores as covariates, and generated Q-Q plot (Fig. S2a). Lambda GC (GC_1000_) was 1.077 (1.025), indicating the low possibility of population substructure. We identified 14 genotyped SNPs with genome-wide significant association (*P* < 5 × 10^−8^) in two loci: chromosome 12q24.11–12 and 20q12 (Fig. S3a and Table S2). To investigate additional susceptibility loci, we performed whole-genome imputation and also generated Q-Q plot (Fig. S4a). 6,007,297 SNPs passed QC of the imputation, and lambda GC (GC_1000_) was 1.083 (1.027). 112 SNPs achieved the genome-wide significance. All these SNPs were located in the same two loci identified by the GWAS using genotyped SNPs (Fig. 1a).

### Stratified GWASs by risk factors

To identify subgroup specific associations, we performed stratified GWASs by the groups of IONFH based on its two major risk factors, i.e. steroid treatment and alcohol drinking. The definitions of IONFH stratification were as follows: Steroid-associated ONFH was defined as having a history of systemic steroid administration, independent of dosage^24^. Alcohol-associated ONFH was defined as having a history of 400 ml or more ethanol consumption per week and absence of systemic steroid administration^25,26^. Patients meeting neither definition were categorized as neither-associated IONFH. As a result, IONFH cases were classified into 1,058 steroid-associated, 351 alcohol-associated and 132 neither-associated ONFHs (Table 1). Among the controls, 3,647 subjects had alcohol drinking history of 400 ml or more ethanol consumption per week. Stratified GWASs were performed in the same way as the GWAS in all IONFH cases. Gender and top 10 principal component scores, which were used in all IONFH cases and controls, were shared as covariates.

GWAS for alcohol-associated ONFH gave similar results to those for IONFH (Fig. 1b and Figs S2–S4b), showing 129 SNPs with genome-wide significance. All SNPs were located within 12q24.11–13 and 20q12. When BBJ controls were stratified based on the alcohol-drinking amount, eight SNPs with genome-wide significance were identified, all of which were located in only the 20q12 locus (Fig. 1c and Figs S2–S4c).

GWAS for steroid-associated ONFH showed two genotyped SNPs in 2q32.3 with genome-wide significance: rs10168266 and rs11889341 (Figs S2–S3d). Imputation analysis showed eight SNPs with genome-wide significance in 2q32.3 and 6p21.32 (Fig. 1d and Fig. S4d). In the 2q32.3 locus, rs13426947 had the smallest *P* value (*P* = 7.05 × 10^−14^) and was in strong LD with rs10168266 and rs11889341 (LD index of *r* ^2^ > 0.8) (Fig. S5). These SNPs were located in the intron of *STAT4* (signal transducer and activator of transcription 4), which was reported to be associated with several autoimmune diseases^27,28^. Especially, rs10168266 was shown to be associated with SLE^27^ and Sjögren’s syndrome^28^. In fact, the association of rs13426947 largely depended on SLE patients (Table 2). The *P* value on steroid-associated ONFH without SLE unsatisfied genome-wide significance (*P* = 0.0138), and the odds ratio decreased considerably (from 1.58 to 1.16). Therefore, the significant association in the 2q32.3 locus for steroid-associated ONFH probably reflected the impact of autoimmune diseases including SLE.

**Table 2:** Association of SNPs within 2q32.3 and 6p21.32 on steroid-associated ONFH considering underlying disease

Case		Control	rs13426947 (2q32.3)				rs9268978 (6p21.32)			
			RAF		OR	*P*	RAF		OR	*P*
			Case	Control			Case	Control		
Steroid-associated ONFH	1,058	59,103	0.338	0.278	1.33	1.63 × 10^−9^	0.112	0.081	1.52	1.16 × 10^−8^
***Underlying disease***										
SLE	433	59,103	0.378	0.278	1.58	9.48 × 10^−11^	0.136	0.081	1.88	1.13 × 10^−9^
Not SLE	625	59,103	0.311	0.278	1.16	0.0138	0.096	0.081	1.26	0.0219

ONFH: osteonecrosis of the femoral head, RAF: risk allele frequency, OR: odds ratio, SLE: systemic lupus erythematosus.

In the 6p21.32 locus, there were four SNPs with genome-wide significance including rs92689786 (chr6: 32434978, *P* = 1.16 × 10^−8^) (Fig. S6). These SNPs were located in HLA region, which is strongly associated with autoimmune diseases including SLE^29^. As is the case with rs13426947 in the 2q32.3 locus, the association of rs92689786 largely depended on SLE patients (Table 2). Therefore, the association in the 6p21.32 locus was also probably confounded by autoimmune diseases including SLE.

In the GWAS of neither-associated ONFH and BBJ control, there were several loci in which SNPs satisfied suggestive association threshold (*P* < 1 × 10^−5^); however, no region satisfied genome-wide level of significance (Fig. 1e and FigS S2–S4e).

### Evaluation of the 12q24.11–12 locus

In the 12q24.11–12 locus, the first and third strongest associated SNPs in the analysis for IONFH were rs3858704 (*P* = 2.97 × 10^−12^) and rs4766566 (*P* = 3.31 × 10^−12^), respectively (Fig. S7). These SNPs were in perfect LD (*r* ^2^ = 1). This locus is already known to be associated with alcohol drinking in East Asians^30,31^. rs4766566 is associated with alcohol drinking behavior in Japanese^30^ and with flushing response and daily maximum drinks in Chinese^31^. Therefore, the association of the locus in IONFH was most likely affected by alcohol drinking.

We checked the association of the 12q24.11–12 locus including rs3858704 in the stratified analyses. The association of rs3858704 was extremely strong in alcohol-associated ONFH (*P* = 1.23 × 10^−21^), while it was not in steroid-associated (*P* = 0.0186) or neither-associated ONFHs (*P* = 0.246) (Table 3 and Figs S8a,c,d). These results indicated that the significant association in the 12q24.11–12 locus reflected the result of alcohol-associated ONFH and alcohol drinking affected the development of IONFH.

**Table 3:** Association of rs3858704 and rs6028718

Phenotype	Case	Control	rs3858704 (12q24.12)			rs6028178 (20q12)				
			RAF		*P*	OR	RAF		*P*	OR
			Case	Control			Case	Control		
IONFH	1,547	59,103	0.726	0.672	2.97 × 10^−12^	1.33	0.588	0.521	7.05 × 10^−14^	1.32
***Stratified analysis based on risk factors***										
Alcohol-associated	351	59,103	0.844	0.672	1.23 × 10^−21^	2.73	0.629	0.521	1.98 × 10^−8^	1.56
Steroid-associated	1,058	59,103	0.691	0.672	0.0186	1.12	0.574	0.521	6.84 × 10^−7^	1.25
Neither-associated	132	59,103	0.696	0.672	0.246	1.17	0.587	0.521	0.0298	1.31
Stratified analysis based on alcohol intake*										
Alcohol-associated	351	3,647	0.844	0.817	0.0404	1.26	0.629	0.519	1.43 × 10^−8^	1.61

*Controls were matched for alcohol-drinking history (400 ml or more ethanol consumption per week). RAF: risk allele frequency, OR: odds ratio, IONFH: idiopathic osteonecrosis of the femoral head.

To examine the effect of alcohol drinking, we matched the alcohol intake between alcohol-associated ONFH cases and BBJ controls by extracting controls having a history of 400 ml or more ethanol consumption per week. As a result, the association in the 12q24 locus was no longer significant (Fig. S8b): the *P* value of rs3858704 became 0.0404. Therefore, the 12q24.11–12 locus was associated with IONFH susceptibility through drinking capacity.

### Evaluation of the 20q12 locus

The most strongly associated SNP in the 20q12 locus was rs6028718 (*P* = 7.05 × 10^−14^) (Table 3 and Fig. S9a). We did not find any secondary associations in this locus conditioned on rs6028718 (Fig. S9b). No association with alcohol drinking or autoimmune diseases including SLE has been reported in this locus.

We checked the association of the locus in each group of IONFH (Table 3 and Figs S10 and S11). In contrast to the 12q24.11–12 locus, the *P* value of rs6028718 in all IONFH (*P* = 7.05 × 10^−14^, OR = 1.32) was far lower than that of any of the subgroup. Its association was significant even in alcohol-associated ONFH (*P* = 1.98 × 10^−8^, OR = 1.56). Although not significant, relatively strong association was observed in steroid-associated ONFH (*P* = 6.84 × 10^−7^, OR = 1.25). In neither-associated ONFH, rs6028718 satisfied nominal *P* value (*P* < 0.05, OR = 1.31) despite the small number of cases. The association in the 20q12 locus remained significant after adjustment of alcohol drinking: rs6028718 had the smallest *P* value (*P* = 1.43 × 10^−8^, OR = 1.61). Therefore, we concluded that the 20q12 locus represented by rs6028718 is IONFH susceptibility locus regardless of alcohol drinking or steroid treatment.

### Identification of LINC01370 as a candidate gene in the 20q12 locus

**Figure 2:**
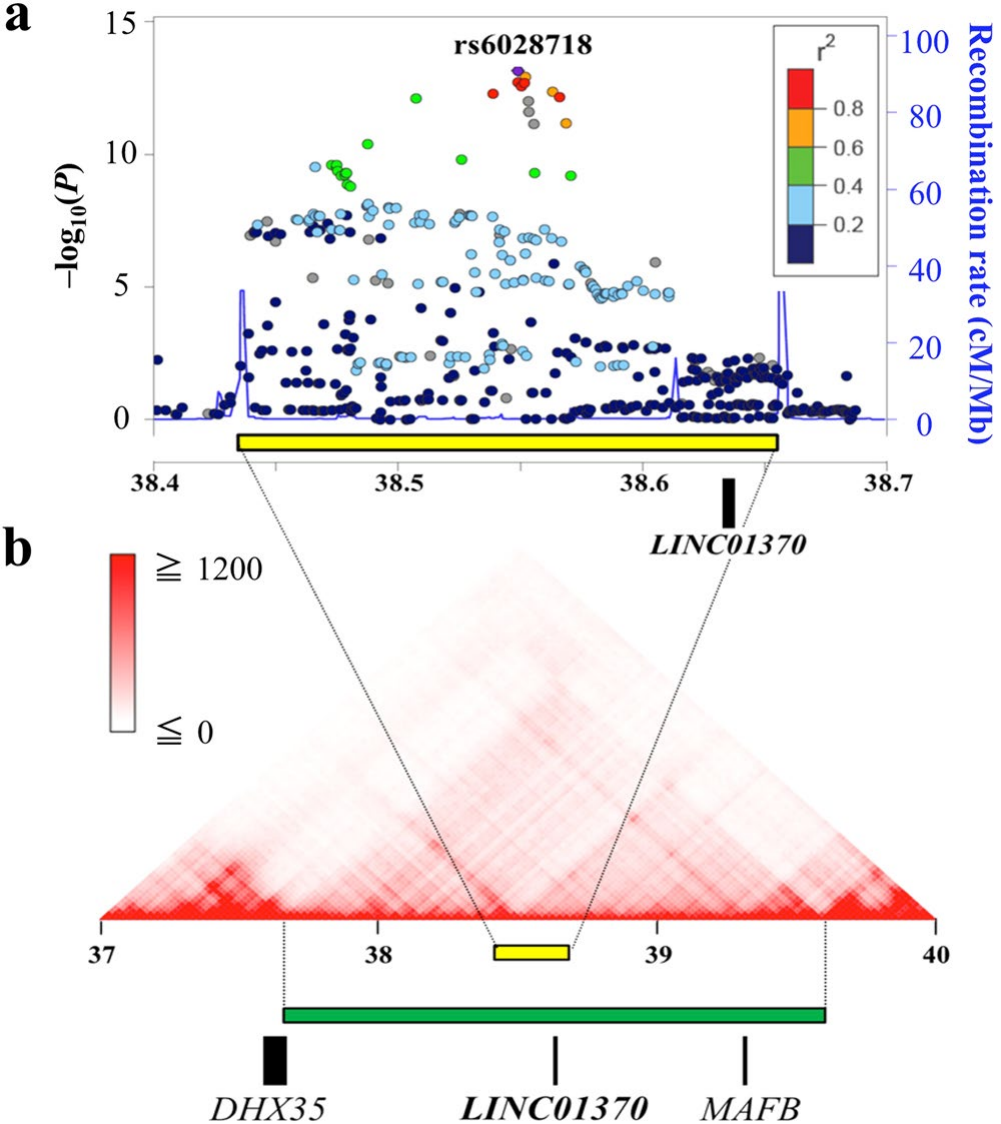
Evaluation of the 20q12 locus (**a**) Regional association plot derived from the GWAS result of imputation analysis for idiopathic osteonecrosis of the femoral head. The region surrounded by distinct peaks of recombination rate (yellow box) was identified as a disease susceptibility locus. Only *LINC01370* was within this locus. The color intensity reflected the extent of linkage disequilibrium index (*r* ^2^) with rs6028718 (in purple). Estimated recombination rates from the hg19/1000 Genomes Project Nov 2014 East Asian reference were shown as light-blue lines. (**b**) The topologically associated domain around the 20q12 locus (green box). Aside from *LINC01370*, this domain contained *DHX35* (DEAH-box helicase 35) and *MAFB* (MAF bZIP transcription factor B).

The 20q12 locus only contained one gene, *LINC01370* (Fig. 2a). *LINC01370* is a long non-coding RNAs, which are defined as non-protein coding transcripts longer than 200 nucleotides and generally show low and tissue specific expression^32^. The GWAS-associated SNPs in the 20q12 locus were located in an extra-genic region, and there were no SNPs in strong LD (*r*2 > 0.8) with lead GWAS SNP in intra-genic regions around the locus (Fig. S12). Therefore, we hypothesized that this locus would be related to transcriptional regulation of nearby or remote genes through the topological association. We evaluated topologically associated domains (TADs) around the locus using Interactive Hi-C Data Browser^33^. In the TAD containing this locus, there were three validated RefSeq genes; *DHX35* (DEAH-box helicase 35), *MAFB* (MAF bZIP transcription factor B) and *LINC01370* (Fig. 2b). We considered these three genes as candidate genes of IONFH susceptibility.

We then examined expression quantitative trait locus (eQTL) data in all available tissues in GTEx^34^ for the association between expression of three candidate genes and 65 genome-wide significant SNPs within the 20q12 locus. The combination of *LINC01370* expression and liver tissue showed the strongest association with genome-wide significant SNPs (Table S3 and Fig. S13). GTEx and FANTOM5^35^ databases showed that *LINC01370* is specifically expressed in liver (Fig. S14). The association of *LINC01370* expression in liver and rs6028718, the lead GWAS SNP in the 20q12 locus, also satisfied nominal *P* value of eQTL (*P* = 0.016). From these eQTL data, *LINC01370* expression in liver was most strongly associated with this locus. In addition, any SNPs in strong LD (*r* ^2^ > 0.8) with lead GWAS SNP were not located in genic region of *DHX35* or *MAFB*, as mentioned before (Fig. S12). Therefore, we think *LINC01730* as the best candidate gene.

### In silico analysis of LINC01370 function

We analyzed the potential functionality of *LINC013*70 *in silico*. We performed pathway enrichment analysis using LncRNA2Function^36^ and Co-LncRNA^37^, both of which use publicly available RNA-Seq data and evaluate co-expression patterns of protein-coding genes for long non-coding RNAs. LncRNA2Function identified 391 co-expressed protein-coding genes with *LINC01370* across 19 human normal tissues. These genes showed significant enrichment associations with lipoprotein in GO terms of cellular components and with lipid and steroid metabolic processes in GO terms of biological process (Table S4). The pathway analysis revealed significant associations with complement and coagulation cascades, as well as lipid and steroid metabolism related pathways (Table S5). About Co-LncRNA, we selected liver as the target tissue for *LINC01370* because of its expression pattern. Two datasets (GSE33294 and GSE63420) were available for analysis of *LINC01370*. 143 co-expressed protein-coding genes with *LINC01370* were commonly identified in both data sets, and they also showed significant enrichment associations with lipid-metabolism related terms and pathways in GO biological process and KEGG (Table S6).

### Pathway analysis for genome-wide association results of imputation data

We performed pathway analysis by MAGENTA^38^ using the GWAS results for IONFH and identified seven significant biological pathways or gene sets associated with IONFH (Table S7). Among them, we found “VEGF signaling” most plausible for the biological pathways associated with IONFH, because IONFH is an ischemic disorder and the VEGF signaling is related to angiogenesis^39^. Some other pathways, such as “Positive regulation of B cell proliferation”, “Chemokine signaling”, and “Fc epsilon RI signaling”, might reflect underlying diseases of steroid treatment, because B cell is related to humoral immunity, chemokines are a family of pro-inflammatory cytokines, and Fc epsilon RI belongs to the immunoglobulin superfamily which plays a role in controlling allergic responses.

## Discussion

We have identified the 20q12 locus with genome-wide significant association on IONFH. Stratified analysis based on the risk factors of IONFH also showed the association of the 20q12 in each group, although their strengths of the associations varied. We think that there are shared disease-susceptibility genes of IONFH regardless of the risk factors. The best candidate gene in the locus is *LINC01370* encoding a type of long non-protein coding RNAs that can have crucial roles in controlling expression of genes during development and differentiation processes^40^. The eQTL analysis suggested that the IONFH-associated variants would increase *LINC01370* expression.

The function of *LINC01370* in IONFH is currently unknown. *In silico* analyses suggested that *LINC01370* could be related to the known plausible pathogenic pathways for IONFH such as blood complement and coagulation, steroid metabolism and lipid metabolism. Notably, lipid metabolism that has been implicated in development of IONFH was indicated by both analyses. Serological studies in SLE showed that high level of total cholesterol^7^ or triglyceride^41^ was associated with steroid-associated ONFH. A rabbit model of steroid-associated ONFH showed enlargement of the bone marrow fat cell^42^, which was also observed in alcohol-treated rabbits^43^. *In vitro* studies showed that steroid and alcohol inhibited osteogenic differentiation and promoted adipogenesis^44–48^.

Among the possible pathways presented by MAGENTA, “VEGF signaling” drew our attention, because the signaling is related to angiogenesis^39^ and the pathology of IONFH is ischemic bone necrosis. Indeed, VEGF protein is expressed in the reparative interface zone adjacent to the necrotic zone in the resected femoral head specimen of IONFH^49,50^. VEGF expression is also observed around necrotic region in a rabbit model of steroid-associated ONFH^51^. These findings suggest that VEGF signaling pathway is closely related to the repairing process of osteonecrosis.

Stratified analyses showed different patterns of association in each group (Fig. 1). The difference might derive from specific ONFH-susceptibility in each group, which could be detected by increasing sample sizes. Stratified analyses also showed genetic backgrounds related to the risk factors of IONFH: steroid treatment and alcohol drinking. About steroid treatment, the 2q32.3 and 6p21.32 loci showed genome-wide significance in the GWAS for steroid-associated ONFH, which probably reflected the underling disease of steroid treatment, especially SLE (Table 2). About alcohol drinking, we detected a significant association of the 12q24 locus with IONFH and alcohol-associated ONFH (Figs S7 and S8). This association disappeared when controls were limited to heavy alcohol drinkers. Therefore, the locus would be associated with IONFH susceptibility through drinking capacity. Thus, stratified analyses based on risk factors are important for adequate evaluation of GWAS for IONFH.

The 12q24 locus on IONFH and alcohol-associated ONFH contains *ALDH2* (alcohol dehydrogenase 2), encoding a major enzyme in alcohol metabolism. rs671 within the gene affects the enzyme activity of ALDH2^52^ and is associated with alcohol drinking behavior in Japanese^30^. Takeuchi *et al*. used 1,462 samples and identified 12 genome-wide significant SNPs within 12q24 region including rs671 (*P* = 1.8 × 10^−30^) and rs4766566 (*P* = 8.4 × 10^−16^). They also showed that rs4766566 was not independent from rs671 by a conditional analysis. In our study, the most significantly associated SNP within the 12q24 locus (rs3858704) is in perfect LD with rs4766566 (*r* ^2^ = 1), suggesting the possible effect of rs671 on the association of the 12q24 locus. Unfortunately, rs671 was excluded from our study because of the significant deviation from HWE (Table S8) and the low imputation quality score (R^2^ = 0.697); however, its calculated association with alcohol-associated ONFH was extremely strong (Table S9). In addition, the conditional analysis adjusting for rs671 genotyping data on IONFH and alcohol-associated ONFH showed no SNPs maintaining genome-wide significance within the 12q24 locus (Fig. S15). These data also supported the theory that the 12q24 locus is associated with IONFH susceptibility through rs671 or drinking capacity.

For steroid-associated ONFH in children with acute lymphoblastic leukemia, four genome-wide significant loci were previously reported^21,22^: rs10989692 (9q31.1), rs79085477 (20q13.31), rs141059755 (8q13.1), and rs1891059 (1q32.3). In our analysis for steroid-associated ONFH, rs10989692 and rs79085477 showed no significant association (Table S10); rs141059755 and rs1891059 were not polymorphic in Japanese. No loci represented by these four SNPs showed significant association (Fig. S16). There are a few possible reasons for the difference in the results between our GWAS and previous reports. First, the allele frequency of each SNP is different by ethnic groups (Table S10). Second, the samples used in previous reports were mostly children with acute lymphoblastic leukemia, while ours were almost all adult and consisted of any diseases requiring steroid treatment. Previous reports covered only the small portion of IONFH because IONFH including steroid-associated ONFH mainly occurs in adult and that SLE is the most frequent underlying disease of steroid-associated ONFH^3,53–55^. Third is the different definition of the case. The case in previous reports was defined by the presence of symptoms: asymptomatic ONFH patients were used as controls. Our case was defined by the presence of radiographic ONFH regardless of symptoms, which is more appropriate in exploring disease susceptibility.

One limitation of the present study is that we could not check the existence or absence of IONFH among BBJ controls. There may be the possibility that some BBJ controls had IONFH. However, we think that the proportion of such samples is very small and thus their effect on the analysis is negligible, because the estimated prevalence of IONFH in Japanese is less than 0.1%^3^ and the samples belonging to the disease categories likely to be related to the risk factors of IONFH were removed. Another limitation is that there is no consensus about definition of IONFH stratification. Currently, it is generally accepted that 400 ml or more ethanol consumption per week is the risk factor of IONFH^25,26^ and that steroid is much stronger risk factor of IONFH than alcohol^56^. Based on the evidence, we stratified IONFH into three groups in this study. Finally, our results lack functional and replication studies. To elucidate the function of IONFH-susceptibility loci, we are now performing molecular biological analysis. For the replication study, international cooperation and meta-analysis are desired, which could enable further identification of IONFH-susceptibility loci.

In conclusion, we performed GWAS and whole genome imputation using 1,602 Japanese IONFH cases and 60,000 controls, and identified the 20q12 locus with genome-wide significance that contained *LINC01370* as a candidate gene for IONFH susceptibility. We also identified the 12q24.11–12 locus associated with IONFH susceptibility through determining the alcohol drinking capacity. Our data would be useful to clarify the yet-to-be-defined etiology and pathophysiology of IONFH. Different patterns of associations in our stratified analyses by known clinical risk factors suggested the presence of group-specific susceptibility genes in each group of IONFH in addition to common susceptibility genes, highlighting the importance of careful phenotyping and good clinical data to deal with genetic heterogeneity of this disease. Further studies are necessary to identify the common and sub-group specific IONFH susceptibility genes.

## Methods

### Subject recruitment

Clinical information and peripheral blood samples from IONFH patients were collected from 23 hospitals in Japan, which were members of the Japanese Research Committee (JRC) on IONFH in the Ministry of Health, Labour and Welfare and their affiliated hospitals. Clinical information collected from the patients consisted of gender, age at time of sample collection, age at onset of symptoms, clinical history, amount of alcohol drinking, and steroid treatment. The underlying disease of steroid treatment was also investigated. For controls, we used genome-wide screening data in the BioBank Japan Project (BBJ)^23^ consisting of samples with 47 diseases. After excluding 13 diseases that were likely to relate to IONFH and its risk factors, we randomly selected 60,000 samples. A written informed consent was obtained from all patients and/or guardians. This study was approved by Institutional Review Boards (IRB) of Kyushu University, RIKEN, and all participating institutes for sample collecting (IRB of Osaka University, Hokkaido University, Showa University, Chiba University, Nagoya University, Niigata University, Keio University, Tokyo Women’s Medical University, Saitama Medical University, Kyoto Prefectural University of Medicine, Saga University, Kanazawa Medical University, Osaka City University, Kyushu Medical Center, Kanazawa University, Juntendo University, Kurume University, Mie University, Kansai Rosai Hospital, University of Occupational and Environmental Health, Hiroshima University, and Kyoto University). All methods were carried out in accordance with the relevant guidelines and regulations. The datasets generated during the current study are available from the corresponding authors on reasonable request.

### Diagnosis and classification of IONFH

The diagnosis of IONFH was based on the criteria of JRC on IONFH^5^. In brief, the criteria consist of five findings: (1) collapse (including crescent sign) of the femoral head without joint space narrowing or acetabular abnormality on X-rays, (2) demarcating sclerosis in the femoral head without joint space narrowing or acetabular abnormality on X-rays, (3) “cold in hot” on bone scans, (4) low-intensity band on T1-weighted magnetic resonance imaging, (5) trabecular and marrow necrosis on histology. IONFH was diagnosed if the patient fulfilled two or more of these five findings and did not have bone tumors or skeletal dysplasias.

### SNP genotyping and quality control (QC) for GWAS

Genomic DNA was extracted from peripheral blood leukocytes using a standard method. For the GWAS, we genotyped case samples using the Illumina HumanOmniExpressExome BeadChip and control samples using the Illumina HumanOmniExpressExome BeadChip or a combination of HumanOmniExpress and HumanExome BeadChips. The samples with genotyping call rate <98% were excluded. QC of genotyped SNPs was then performed using a standard method: we excluded SNPs (1) with a call rate <99% in either case or control, (2) deviating from Hardy-Weinberg equilibrium (HWE) (*P* < 1 × 10^−6^ in controls), (3) with a minor allele frequency (MAF) <1% (in total) including monomorphic SNPs. Only SNPs within autosomal chromosomes were included in the subsequent analyses.

### Sample QC for GWAS

(1) We evaluated cryptic relatedness by calculating estimates of pairwise IBD (PI_HAT) by PLINK1.9^57^, and removed the samples until the pairs with PI_HAT > 0.1 disappeared. (2) We performed principal component analysis (PCA) by PLINK1.9 with four populations from HAPMAP3^58^: Europeans (CEU), Africans (YRI), Japanese (JPT), and Han Chinese (CHB). Then we removed outliers who did not belong to the East Asia cluster (Fig. S1). SNPs not in human leukocyte antigen (HLA) region (chr 6: 28.5~33.5 Mb) were pruned based on linkage disequilibrium (LD) and used for PCA. Scatterplot was generated using top three principal component scores by R. (3) We estimated gender for each sample by calculating the percentage of X chromosome heterogeneity (pXhet), and removed the gender-mismatched samples between genotype and clinical information. The exclusion thresholds of pXhet were >0.05 for male and <0.10 for female. (4) We also removed the subjects who had received bone marrow transplantation for blood diseases.

### GWAS

We performed logistic regression analysis using PLINK1.9 and evaluated the association of each genotyped SNP that passed QC. As covariates, we used gender and top ten principal component scores, which were re-calculated using only quality-controlled samples. We determined the number of principal components to use as covariates by considering lambda GC and GC_1000_. We also constructed a quantile-quantile (Q-Q) plot of observed *P*-values against expected *P*-values and Manhattan plot by R. Then we generated regional association plots using LocusZoom^59^.

### Imputation

We performed phasing (haplotype estimation) with SHAPEITv2^60^ and imputation of non-genotyped SNPs with Minimac3^61^. We used the phase 3 data (version 5) of cosmopolitan populations from 1000 Genomes Project^62^ as a reference. Since imputation errors are more serious when allele frequencies are low, we excluded SNPs with (1) MAF of < 1% and (2) a low quality of imputation (Minimac software quality score of *R* ^2^ < 0.9). After these QC of imputed SNP data, association tests were performed for dosage with Mach2dat^63^. Gender and top ten principal component scores mentioned above were used as covariates.

### Conditional analysis by top SNPs

For the genomic region including SNPs with genome-wide significance and surrounded by distinct peaks of recombination rate (>20 cM/Mb), we also carried out conditional analysis to find out secondary associations after conditioning on the lead SNP within the region.

### Stratified analysis based on risk factors

IONFH cases were stratified into three groups based on risk factors^3^, and GWASs were performed in the same way as done in all IONFH cases. Gender and top ten principal component scores, which were calculated in all IONFH cases and controls, were shared as covariates.

To examine the effect of alcohol drinking on the GWAS of alcohol-associated ONFH, BBJ controls with an alcohol-drinking history of 400 ml or more ethanol consumption per week were extracted. This amount of alcohol drinking was in accordance with the criteria of alcohol-associated ONFH in this study. We performed GWAS in the same way as others.

### Characterization of the 20q12 locus

We defined the disease-susceptibility locus as the region containing the significantly associated SNPs and surrounded by distinct peaks of recombination rate (>20 cM/Mb). We evaluated topologically associated domains (TADs) around the locus using Interactive Hi-C Data Browser^33^. Then we examined the expression of the candidate genes in the disease-susceptibility locus and TAD using the gene-expression databases, such as GTEx^34^ and FANTOM5^35^. We also checked the data of expression quantitative trait locus (eQTL) for the genes using GTEx. To investigate the function of *LINC01370*, we performed pathway enrichment analysis using LncRNA2Function^36^ and Co-LncRNA^37^, both of which use publicly available RNA-Seq data and evaluate co-expression patterns of protein-coding genes for long non-coding RNAs. About Co-LncRNA, we selected liver as the target tissue for *LINC01370* because of its expression pattern.

### Pathway analysis for genome-wide association results of imputation data

Complex disorders may result from the accumulation of the mild effects of genetic variants within biological pathways^64^. Therefore, pathway analysis for GWAS results was conducted using MAGENTA^38^ to discover biological pathways or gene sets associated with IONFH. Briefly, the steps of MAGENTA analysis were as follows: (1) SNPs used in the analysis for IONFH were mapped with genes which were located at a predetermined boundary (SNPs within HLA region were excluded); (2) Gene scores were ranked based on the regional SNP *P*-values, and the best SNP *P*-values were determined; (3) Gene scores were corrected for confounders, such as gene size and LD-related properties; and (4) Gene-set enrichment *P*-values were determined by analyzing the gene sets enriched with highly ranked gene scores and the selected biological pathway or gene sets. 95th percentile was set as threshold for gene-level significance. False discovery rate (FDR) less than 0.05 was set as threshold for the significance of a gene-enrichment pathway.

## Electronic supplementary material

Supplementary information
