# Supplementary material for: Genome-wide Association Study of Idiopathic Osteonecrosis of the Femoral Head
Source: Sci Rep. 2017 Nov 8;7:15035. doi: 10.1038/s41598-017-14778-y (PMC5678103; doi:10.1038/s41598-017-14778-y)
Supplement: Supplementary file 1 — Supplementary information [file 41598_2017_14778_MOESM1_ESM.pdf]

SUPPLEMENTARY INFORMATION

TITLE:

Genome-wide Association Study of Idiopathic Osteonecrosis of the Femoral Head

Yuma Sakamoto[1, 2], Takuaki Yamamoto[3], Nobuhiko Sugano[4], Daisuke Takahashi[5], Toshiyuki Watanabe[6], Takashi Atsumi[7], Junichi Nakamura[8], Yukiharu Hasegawa[9], Koichi Akashi[10], Ichiei Narita[11], Takeshi Miyamoto[12], Tsutomu Takeuchi[13], Katsunori Ikari[14], Koichi Amano[15], Atsuhiro Fujie[12], Toshikazu Kubo[16], Yoshifumi Tada[17], Ayumi Kaneuji[18], Hiroaki Nakamura[19], Tomoya Miyamura[20], Tamon Kabata[21], Ken Yamaji[22], Takahiro Okawa[23], Akihiro Sudo[24], Kenji Ohzono[25], Yoshiya Tanaka[26], Yuji Yasunaga[27], Shuichi Matsuda[28], Yuuki Imai[29], Japanese Research Committee on Idiopathic Osteonecrosis of the Femoral Head*, Masato Akiyama[30], Michiaki Kubo[31], Yoichiro Kamatani[30], Yukihide Iwamoto[32], Shiro Ikegawa[2]

[1]Department of Orthopaedic Surgery, Graduate School of Medical Sciences, Kyushu University, 3-1-1 Maidashi, Higashi-ku, Fukuoka-city, Fukuoka, 812-8582, Japan
[2]Laboratory for Bone and Joint Diseases, RIKEN Center for Integrative Medical Sciences, 4-6-1 Shirokanedai, Minato-ku, Tokyo, 108-8639, Japan
[3]Department of Orthopaedic Surgery, Faculty of Medicine, Fukuoka University, 7-45-1 Nanakuma, Jonan-ku, Fukuoka-city, Fukuoka, 814-0180, Japan
[4]Department of Orthopaedic Medical Engineering, Osaka University Graduate School of Medicine, 2-2 Yamadaoka, Suita-city, Osaka, 565-0871, Japan
[5]Department of Orthopaedic Surgery, Hokkaido University Graduate School of Medicine, Kita 15, Nishi 7, Kita-ku, Sapporo-city, Hokkaido, 060-8638, Japan
[6]Division of Rheumatology, Endocrinology and Nephrology, Hokkaido University Graduate School of Medicine, Kita 15, Nishi 7, Kita-ku, Sapporo-city, Hokkaido, 060-8638, Japan
[7]Department of Orthopaedic Surgery, Fujigaoka Hospital, Showa University School of Medicine, 1-30 Fujigaoka, Aoba-ku, Yokohama-city, Kanagawa, 227-8501, Japan
[8]Department of Orthopaedic Surgery, Graduate School of Medicine, Chiba University, 1-8-1 Inohana, Chuo-ku, Chiba-city, Chiba, 260-8677, Japan
[9]Department of Orthopaedic Surgery, Nagoya University Graduate School of Medicine, 65 Tsurumai-cho, Showa-ku, Nagoya-city, Aichi, 466-8550, Japan
[10]Department of Medicine and Biosystemic Science, Kyushu University Graduate School of Medical Sciences, 3-1-1 Maidashi, Higashi-ku, Fukuoka-city, Fukuoka, 812-8582, Japan
[11]Division of Clinical Nephrology and Rheumatology, Kidney Research Center, Niigata University Graduate School of Medical and Dental Sciences, 1-757 Chuo-ku, Niigata-city, Niigata, 951-8510, Japan
[12]Department of Orthopedic Surgery, Keio University School of Medicine, 35 Shinano-machi, Shinjuku-ku, Tokyo, 160-8582, Japan

[13]Division of Rheumarology, Department of Internal Medicine, Keio University School of Medicine, 35 Shinano-machi, Shinjuku-ku, Tokyo, 160-8582, Japan
[14]Institute of Rheumatology, Tokyo Women's Medical University, 10-22 Kawada-cho, Shinjuku-ku, Tokyo, 162-0054, Japan
[15]Department of Rheumatology and Clinical Immunology, Saitama Medical Center, Saitama Medical University, 1981 Kamoda, Kawagoe-city, Saitama, 350-8550, Japan
[16]Department of Orthopaedics, Graduate School of Medical Science, Kyoto Prefectural University of Medicine, 465 Kajii-cho, Kawaramachi-Hirokoji, Kamigyo-ku, Kyoto-city, Kyoto, 602-8566, Japan
[17]Department of Rheumatology, Faculty of Medicine, Saga University, 5-1-1 Nabeshima, Saga-city, Saga, 849-8501, Japan
[18]Department of Orthopaedic Surgery, Kanazawa Medical University, 1-1 Daigaku, Uchinada-machi, Kahoku-gun, Ishikawa, 920-0293, Japan
[19]Department of Orthopaedic Surgery, Osaka City University Graduate School of Medicine, 1-4-3, Asahi-machi, Abeno-ku, Osaka-city, Osaka, 545-8585, Japan
[20]Department of Internal Medicine and Rheumatology, National Hospital Organization Kyushu Medical Center, 1-8-1 Jigyohama, Chuo-ku, Fukuoka-city, Fukuoka, 810-8563, Japan
[21]Department of Orthopaedic Surgery, Graduate School of Medical Science, Kanazawa University, Takara-machi 13-1, Kanazawa-city, Ishikawa, 920-8641, Japan
[22]Department of Internal Medicine and Rheumatology, Juntendo University School of Medicine, 2-1-1 Hongo, Bunkyo-ku, Tokyo, 113-8421, Japan
[23]Department of Orthopaedic Surgery, Kurume University Medical Center, 155-1 Kokubu-machi, Kurume-city, Fukuoka, 839-0863, Japan
[24]Department of Orthopaedic Surgery, Mie University Graduate School of Medicine, 2-174 Edobashi, Tsu-city, Mie, 514-8507, Japan
[25]Department of Orthopaedic Surgery, Kansai Rosai Hospital, 1-69 Inabasou 3-chome, Amagasaki-city, Hyogo, 660-8511, Japan
[26]The First Department of Internal Medicine, University of Occupational and Environmental Health, School of Medicine, 1-1 Iseigaoka, Yahatanishi-ku, Kitakyushu-city, Fukuoka, 807-8555, Japan
[27]Department of Orthopaedic Surgery, Hiroshima Prefectural Rehabilitation Center, Taguchi 295-3, Saijo-cho, Higashi-Hiroshima-city, Hiroshima, 739-0036, Japan
[28]Department of Orthopaedic Surgery, Graduate School of Medicine, Kyoto University, 54 Kawahara-cho, Shogoin, Sakyo-ku, Kyoto-city, Kyoto, 606-8507, Japan
[29]Division of Integrative Pathophysiology, Proteo-Science Center, Ehime University Graduate School of Medicine, Shitsukawa, Toon-city, Ehime, 791-0295, Japan
[30]Laboratory for Statistical Analysis, RIKEN Center for Integrative Medical Sciences, 1-7-22 Suehiro-cho, Tsurumi-ku, Yokohama-city, Kanagawa, 230-0045, Japan
[31]RIKEN Center for Integrative Medical Sciences, 1-7-22 Suehiro-cho, Tsurumi-ku, Yokohama-city, Kanagawa, 230-0045, Japan
[32]Department of Orthopaedic Surgery, Kyushu Rosai Hospital, 1-1 Sonekita-machi, Kokuraminami-ku, Kitakyushu-city, Fukuoka, 800-0229, Japan

Supplemental Figure 1. Principal component analysis of GWAS samples with HAPMAP four populations

Scatter plots of **(a)** principal component 1 (PC1) and PC2, **(b)** PC1 and PC3, **(c)** PC2 and PC3. In total, there were four outliers in patients with idiopathic osteonecrosis of the femoral head (IONFH), who deviated from East Asia cluster (red box). Four HAPMAP3 populations (JPT, CHB, CEU, YRI) were referred and each group was plotted in different color. BBJ: BioBank Japan, JPT: Japanese in Tokyo (Japan), CHB: Han Chinese in Beijing (China), CEU: Utah residents with Northern and Western European ancestry from the CEPH collection, YRI: Yoruba in Ibadan (Nigeria).

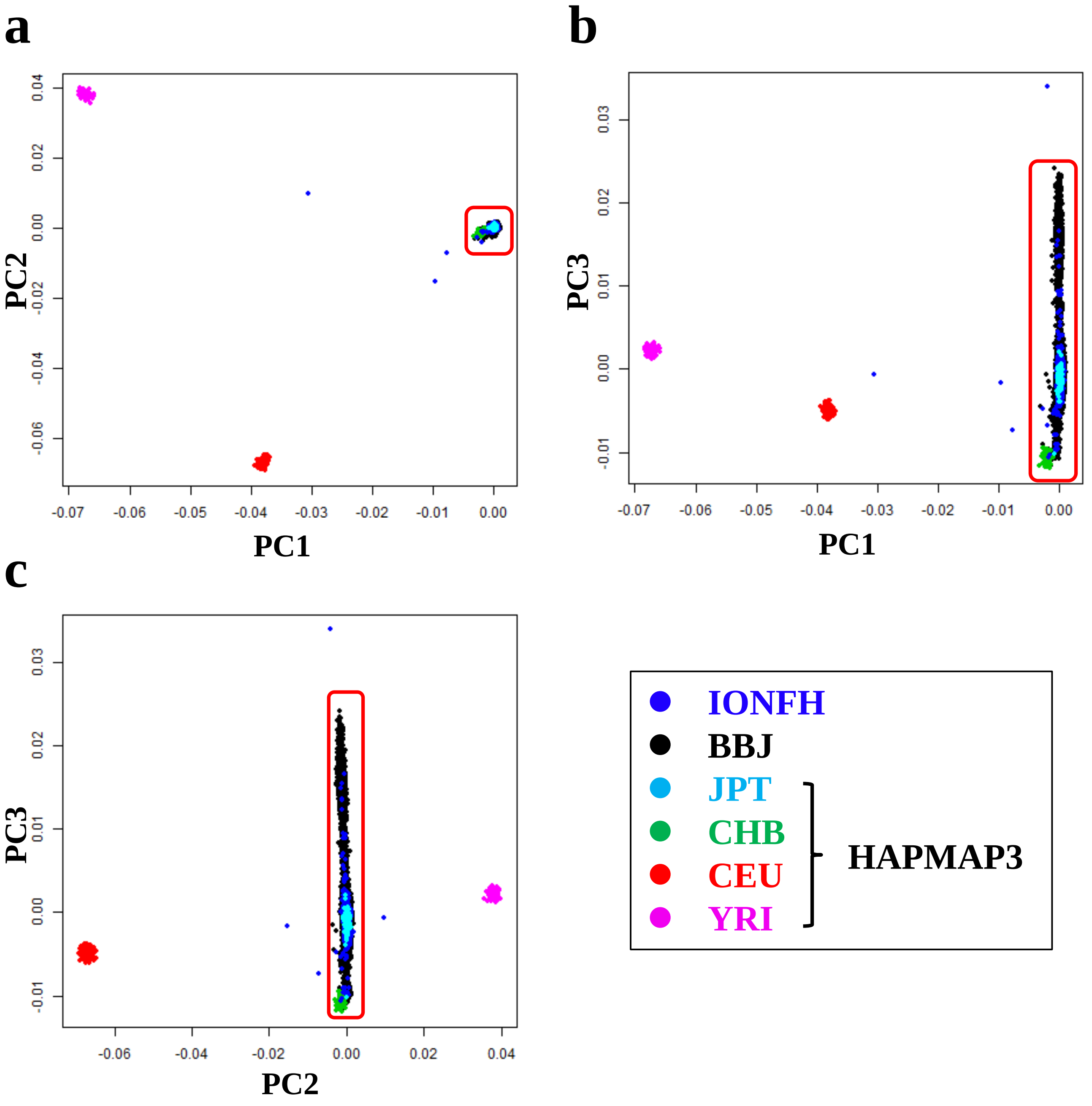

Supplemental Figure 2. Quantile-Quantile plots of genotyped data

(a) Idiopathic osteonecrosis of the femoral head vs BioBank Japan (BBJ), **(b)** Alcohol-associated osteonecrosis of the femoral head (ONFH) vs BBJ, **(c)** Alcohol-associated ONFH vs BBJ of heavy drinker (400 ml/day or more ethanol consumption), **(d)** Steroid-associated ONFH vs BBJ, and **(e)** Neither-associated ONFH vs BBJ. Lambda GCs were **(a)** 1.077, **(b)** 1.039, **(c)** 1.043, **(d)** 1.047, and **(e)** 1.002, respectively.

a

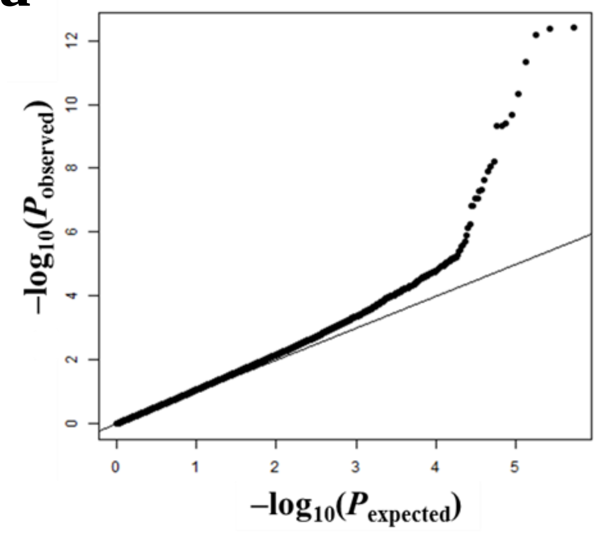

b

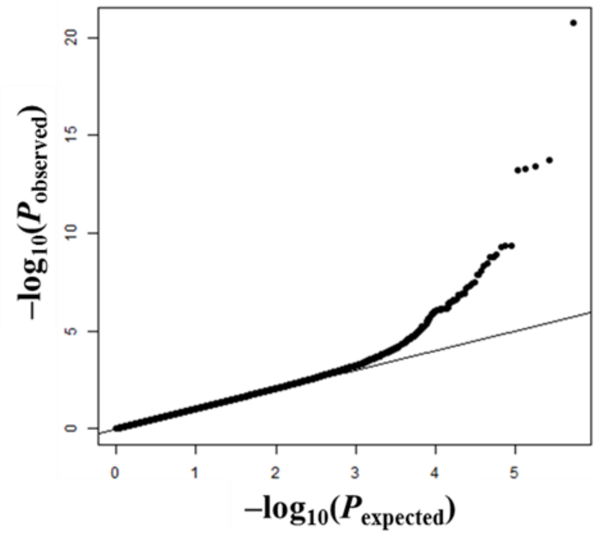

c

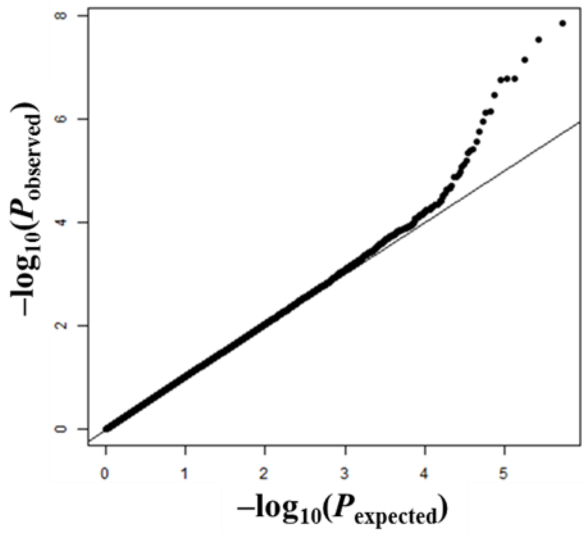

d

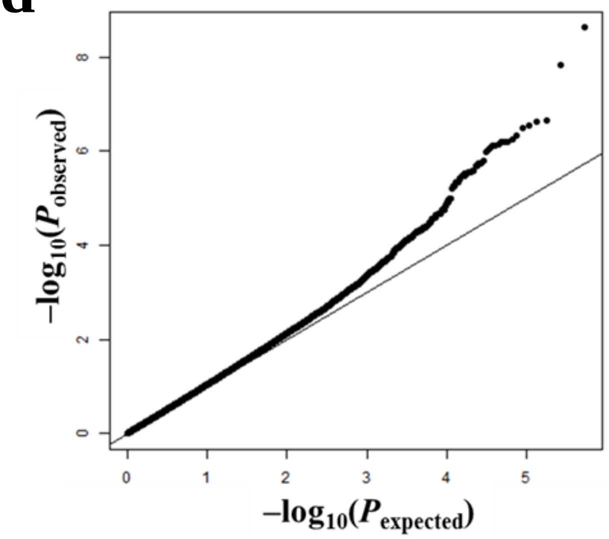

e

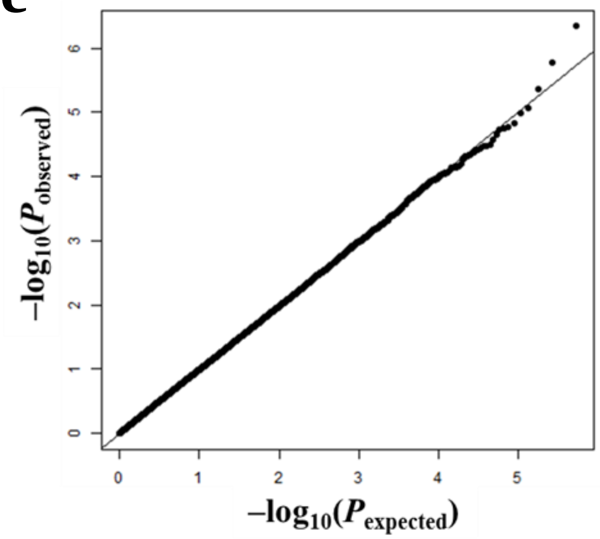

Supplemental Figure 3. Manhattan plots of genotyped data

(a) Idiopathic osteonecrosis of the femoral head vs BioBank Japan (BBJ), **(b)** Alcohol-associated osteonecrosis of the femoral head (ONFH) vs BBJ, **(c)** Alcohol-associated ONFH vs BBJ of heavy drinker (400 ml/day or more ethanol consumption), **(d)** Steroid-associated ONFH vs BBJ, and **(e)** Neither-associated ONFH vs BBJ. The SNPs with genome-wide significance were identified in **(a)** 12q24.11-12, **(b)** 12q24.11-13 and 20q12, **(c)** 20q12, and **(d)** 2q32.3. The red and blue line represented the threshold of genome-wide significance (P = 5 x 10^{-8}) and suggestive association threshold (P = 1 x 10^{-5}), respectively.

a

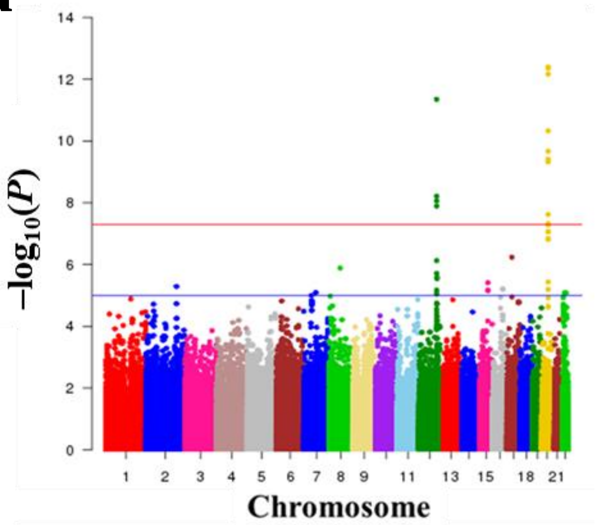

b

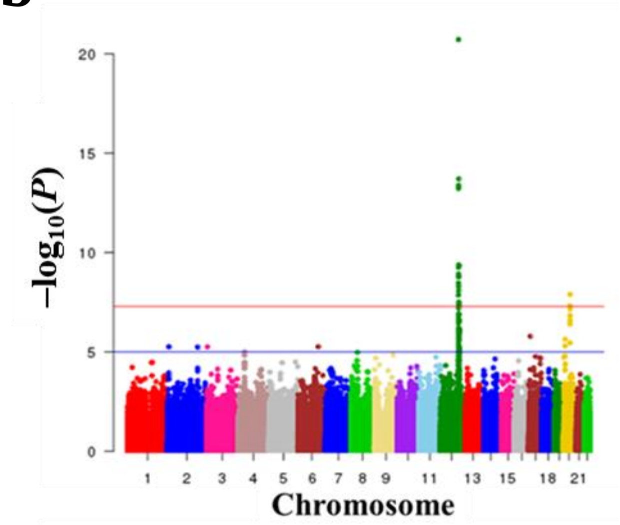

c

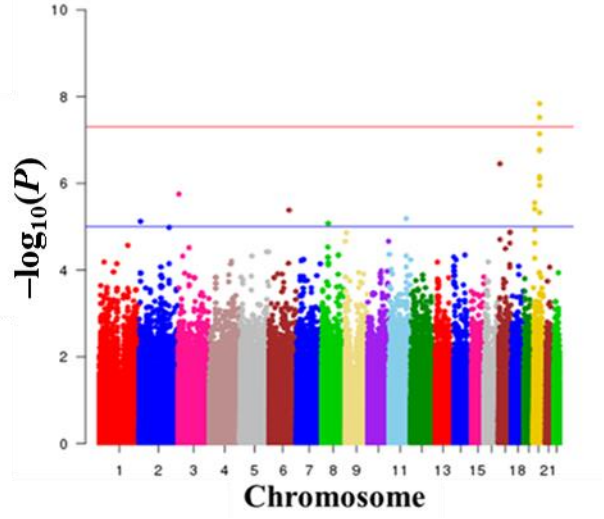

d

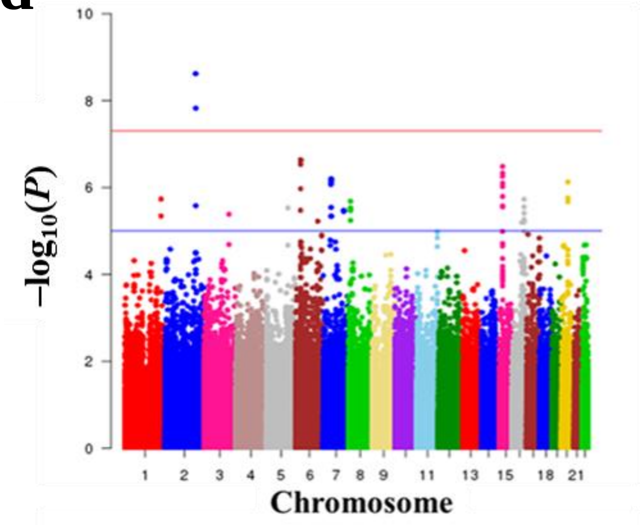

e

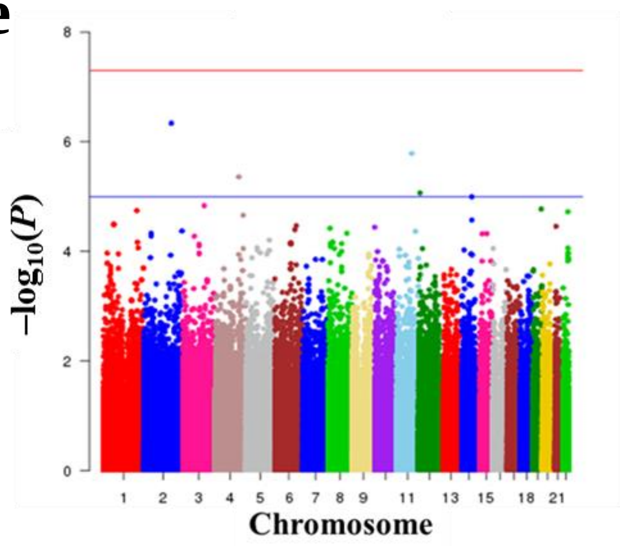

Supplemental Figure 4. Quantile-Quantile plots of imputed data

(a) Idiopathic osteonecrosis of the femoral head vs BioBank Japan (BBJ), **(b)** Alcohol-associated osteonecrosis of the femoral head (ONFH) vs BBJ, **(c)** Alcohol-associated ONFH vs BBJ of heavy drinker (400 ml/day or more ethanol consumption), **(d)** Steroid-associated ONFH vs BBJ, and **(e)** Neither-associated ONFH vs BBJ. Lambda GCs were **(a)** 1.083, **(b)** 1.048, **(c)** 1.052, **(d)** 1.041, and **(e)** 1.006, respectively.

a

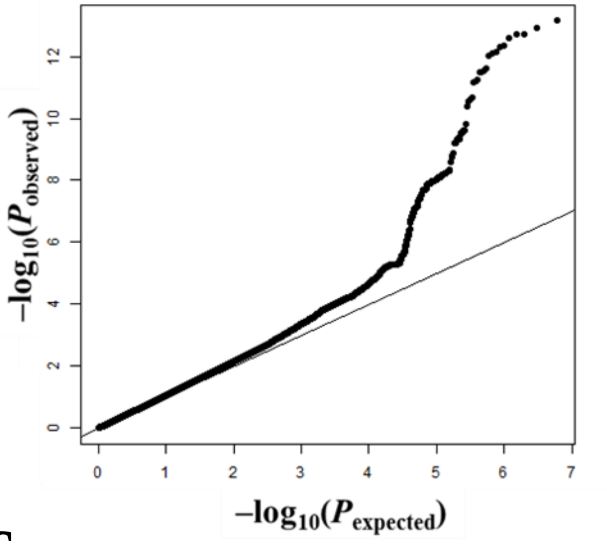

b

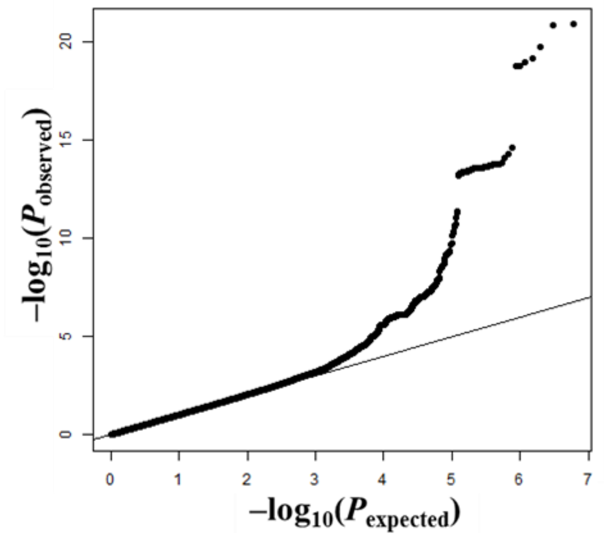

c

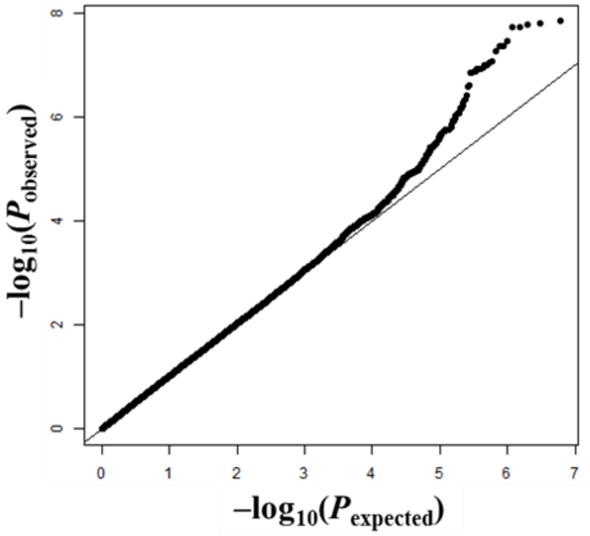

d

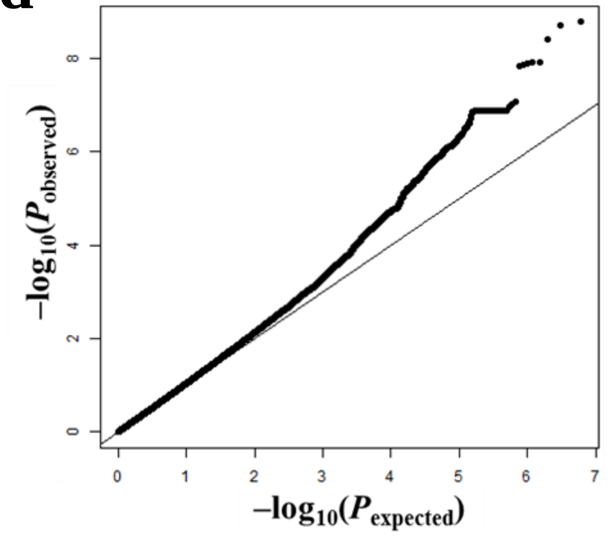

e

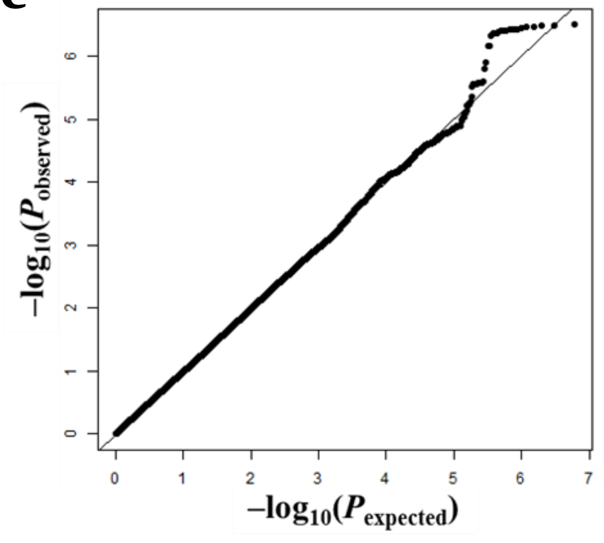

Supplemental Figure 5. Regional association plots of the 2q32.3 locus on the analysis for steroid-associated osteonecrosis of the femoral head

(a) genotyped and **(b)** imputed SNPs. The red line represented the threshold of genome-wide significance (P = 5 x 10^{-8}). The color intensity reflected the extent of linkage disequilibrium index (r^2) with the most strongly associated SNP (in purple). Estimated recombination rates from the hg19/1000 Genomes Project Nov 2014 East Asian reference were shown as light-blue lines.

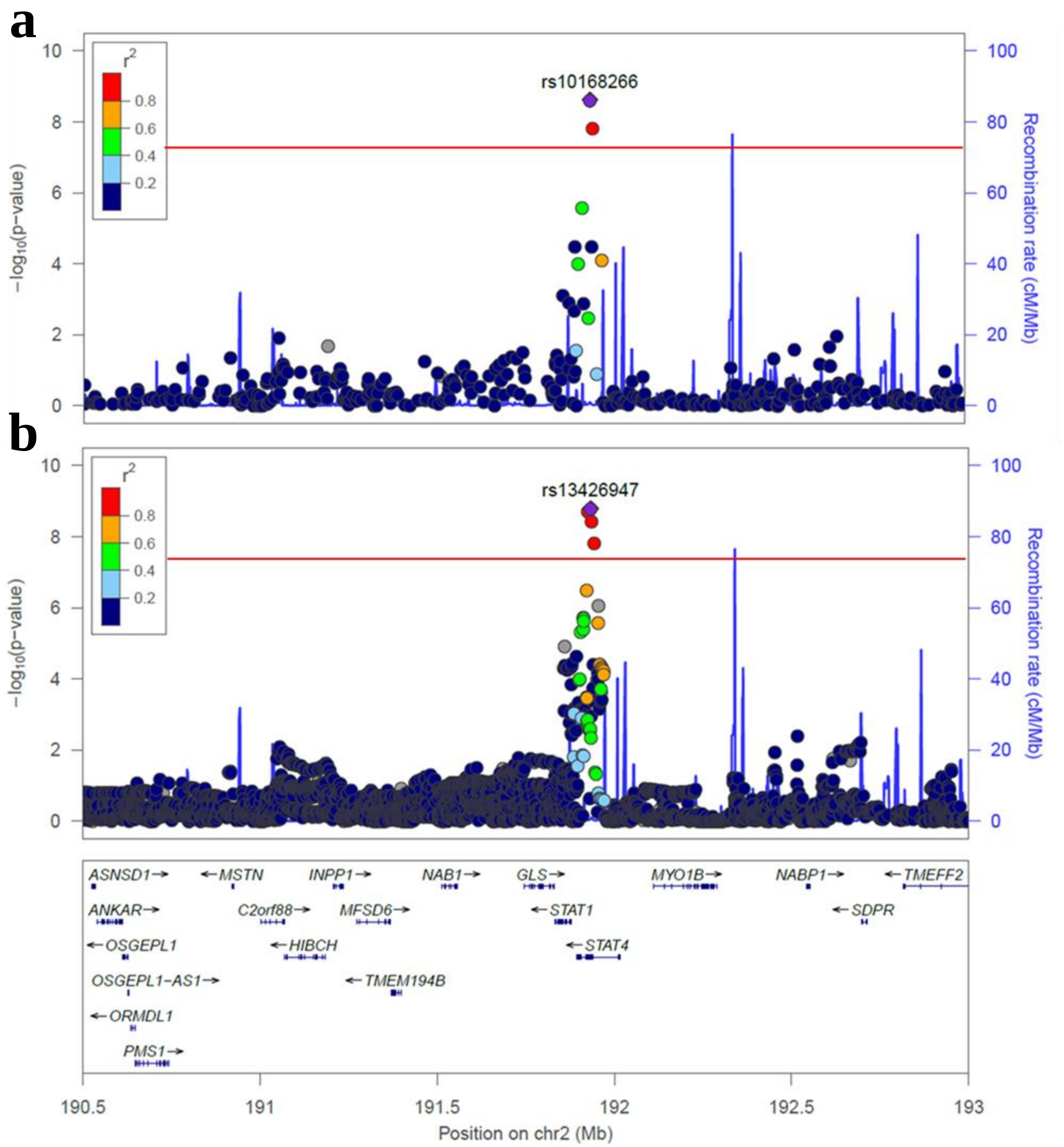

Supplemental Figure 6. Regional association plot of the 6p21.32 locus for steroid-associated osteonecrosis of the femoral head

Four genome-wide significant SNPs including rs92689786 and rs142354442 were located in the major histocompatibility complex region. When adjusting for rs9268978, the genome-wide significance in this locus disappeared (data not shown). The red line represented the threshold of genome-wide significance (P = 5 x 10^{-8}). The color intensity reflected the extent of linkage disequilibrium index (r^2) with rs9268978 (in purple). Estimated recombination rates from the hg19/1000 Genomes Project Nov 2014 East Asian reference were shown as light-blue lines.

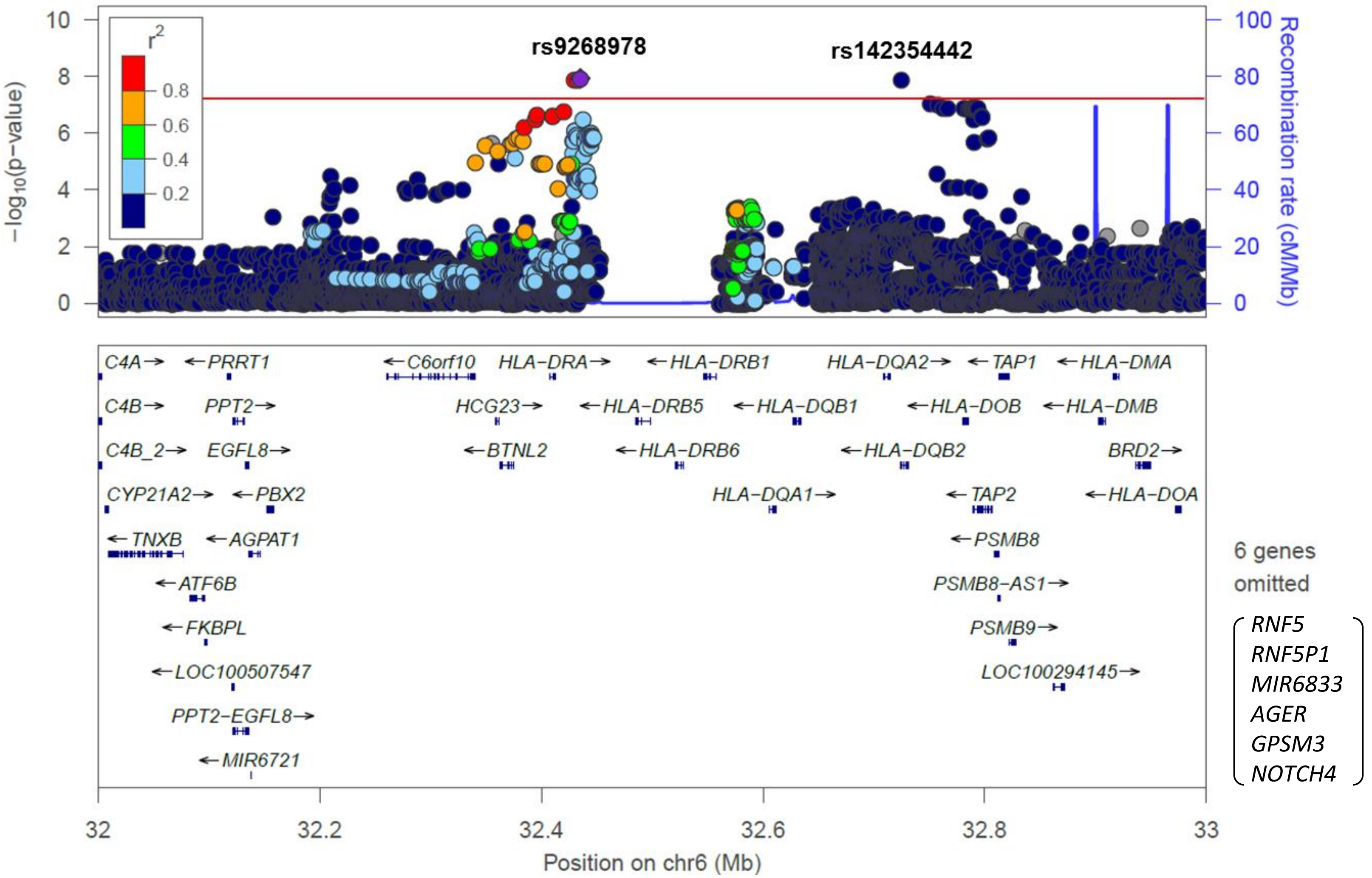

Supplemental Figure 7. Regional association plot of the 12q24 locus for idiopathic osteonecrosis of the femoral head

rs3858704 was the top SNP in this locus (P = 2.97 x 10^{-12}). The red line represented the threshold of genome-wide significance (P = 5 x 10^{-8}). The color intensity reflected the extent of linkage disequilibrium index (r^2) with rs3858704 (in purple). Estimated recombination rates from the hg19/1000 Genomes Project Nov 2014 East Asian reference were shown as light-blue lines.

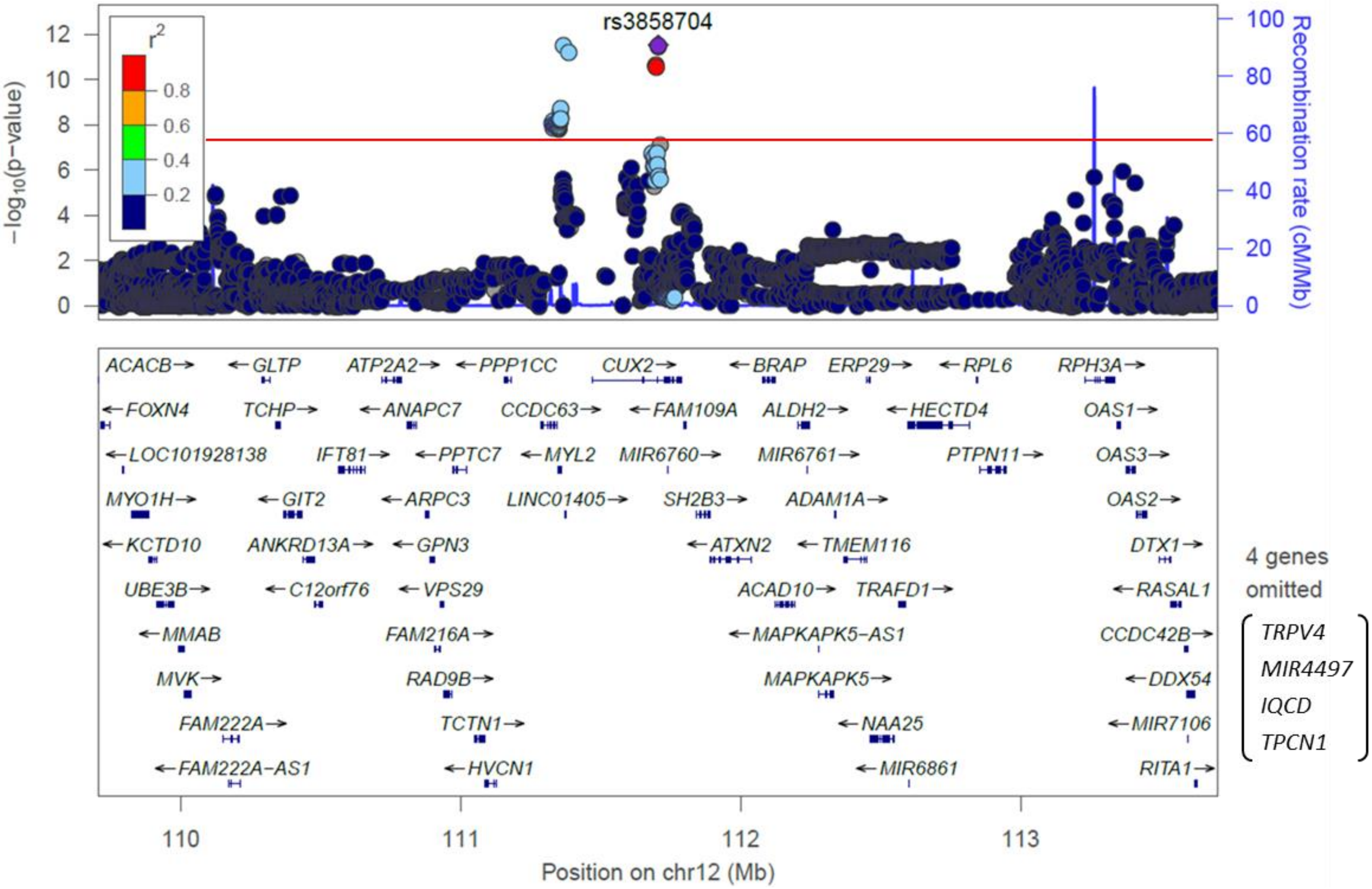

Supplemental Figure 8. Regional association plots of the 12q24 locus for each stratified analysis

(a) Alcohol-associated osteonecrosis of the femoral head (ONFH) vs BioBank Japan (BBJ), **(b)** Alcohol-associated ONFH vs BBJ of heavy drinker (400 ml/day or more ethanol consumption), **(c)** Steroid-associated ONFH vs BBJ, and **(d)** Neither-associated ONFH vs BBJ. **(a)** A strong association was found on Alcohol-associated ONFH. **(b)** When the case and control were matched by alcohol intake, the association on Alcohol-associated ONFH disappeared**. (c, d)** The association was not recognized on Steroid-associated or Neither-associated ONFH. The red line represented the threshold of genome-wide significance ($P = 5 \times 10^{-8}$). The color intensity reflected the extent of linkage disequilibrium index (r^2) with rs3858704 (in purple). Estimated recombination rates from the hg19/1000 Genomes Project Nov 2014 East Asian reference were shown as light-blue lines.

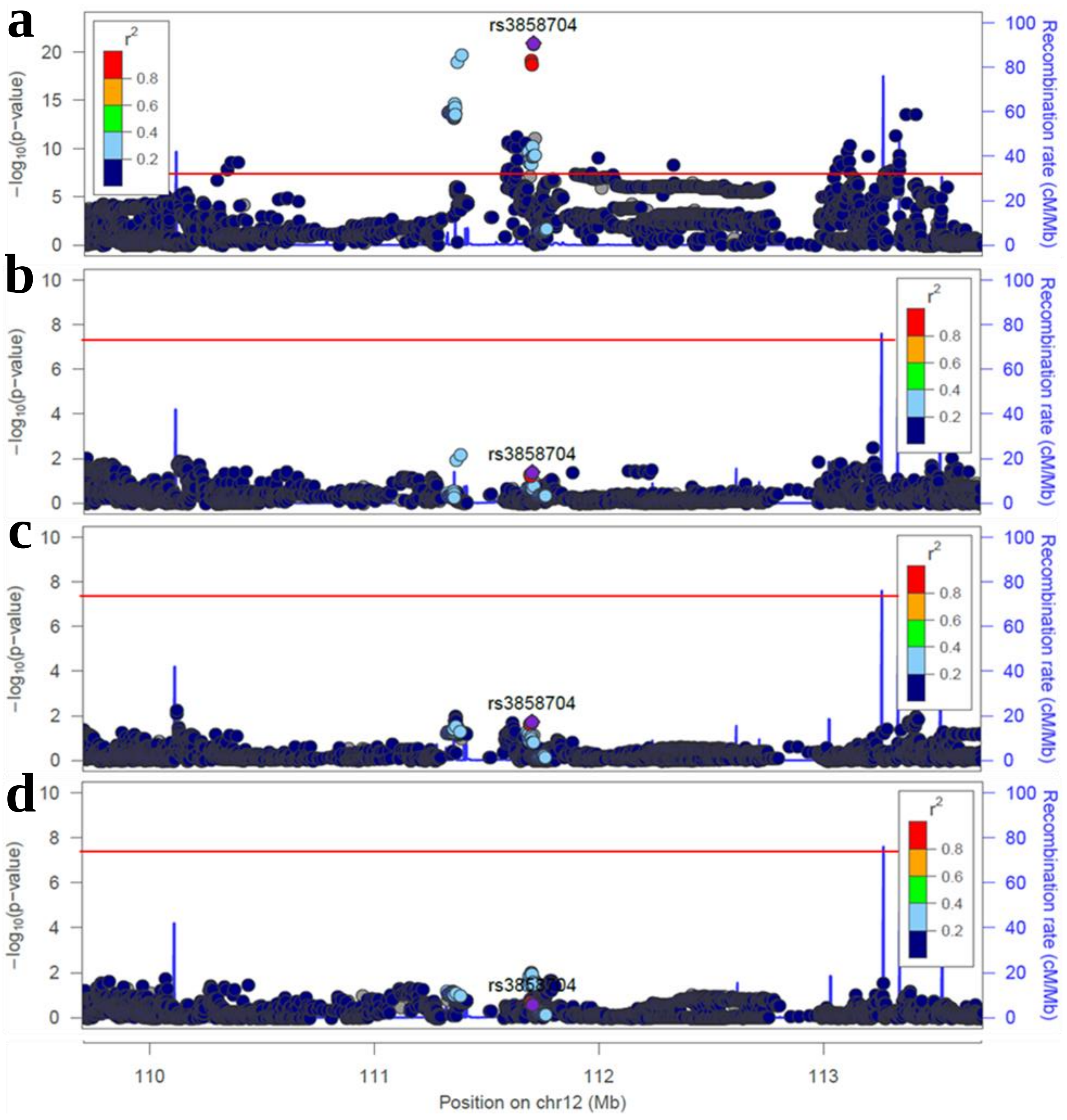

Supplemental Figure 9. Conditional analysis of the 20q12 locus for idiopathic osteonecrosis of the femoral head

The regional association plots **(a)** before and **(b)** after adjusting for rs6028718. **(a)** rs6028718 showed the most significant association (P = 7.05 x 10^{-14}). **(b)** No SNP maintained genome-wide significance (red line: P = 5 x 10^{-8}) when adjusting for rs6028718. The color intensity reflected the extent of linkage disequilibrium index (r^2) with rs6028718 (in purple). Estimated recombination rates from the hg19/1000 Genomes Project Nov 2014 East Asian reference were shown as light-blue lines.

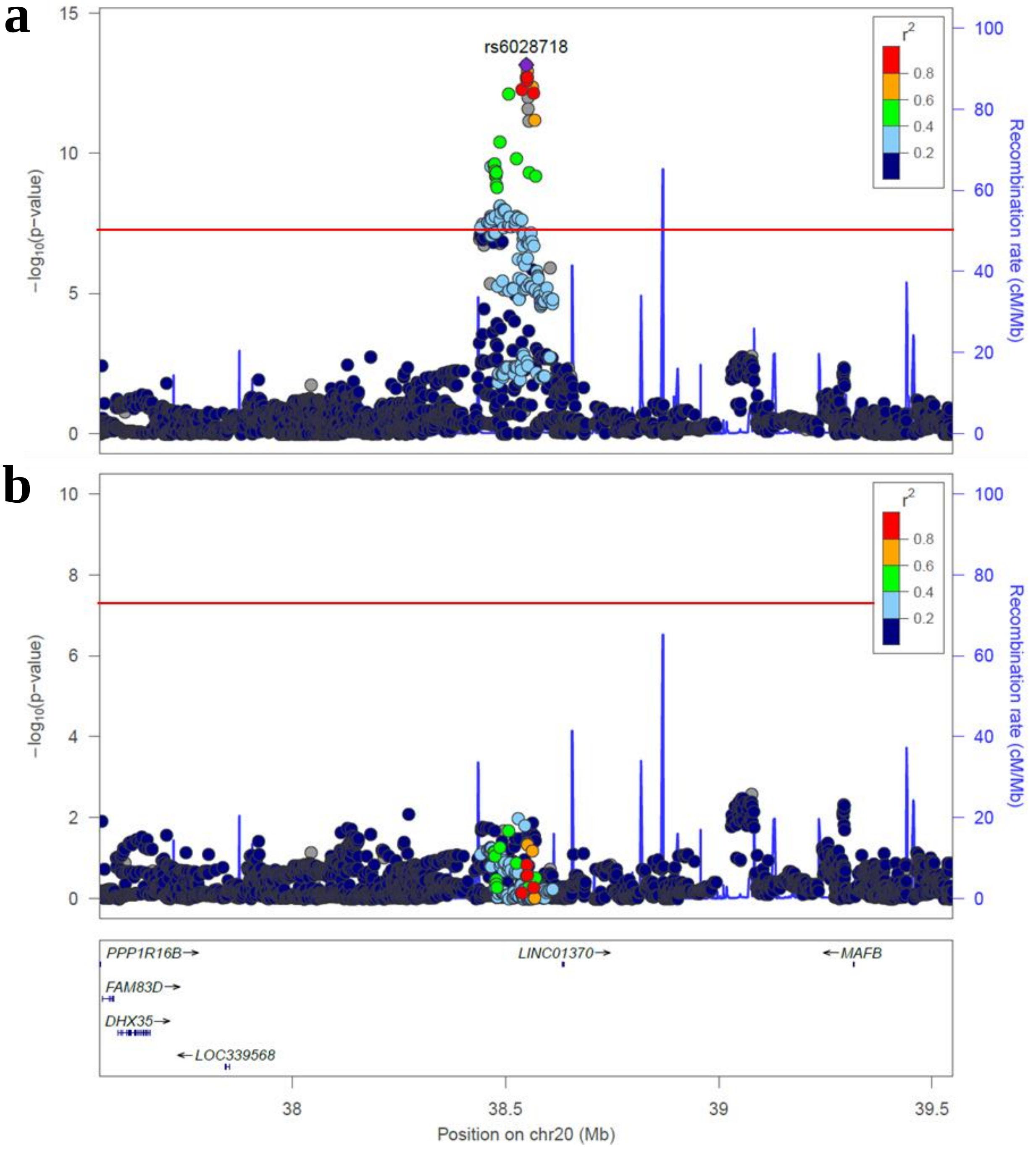

Supplemental Figure 10. Regional association plots of the 20q12 locus for each stratified analysis

(a) Alcohol-associated osteonecrosis of the femoral head (ONFH) vs BioBank Japan (BBJ), **(b)** Alcohol-associated ONFH vs BBJ of heavy drinker (400 ml/day or more ethanol consumption), **(c)** Steroid-associated ONFH vs BBJ, and **(d)** Neither-associated ONFH vs BBJ. **(a)** The genome-wide significant association (red line: $P = 5 \times 10^{-8}$) was found on Alcohol-associated ONFH. **(b)** This association was still significant when case-control was matched by alcohol intake. **(c)** Although not significant, strong association was observed on Steroid-associated ONFH. **(d)** On Neither-associated ONFH, SNPs in this locus satisfied at least nominal P value ($P < 0.05$). The color intensity reflected the extent of linkage disequilibrium index (r^2) with rs6028718 (in purple). Estimated recombination rates from the hg19/1000 Genomes Project Nov 2014 East Asian reference were shown as light-blue lines.

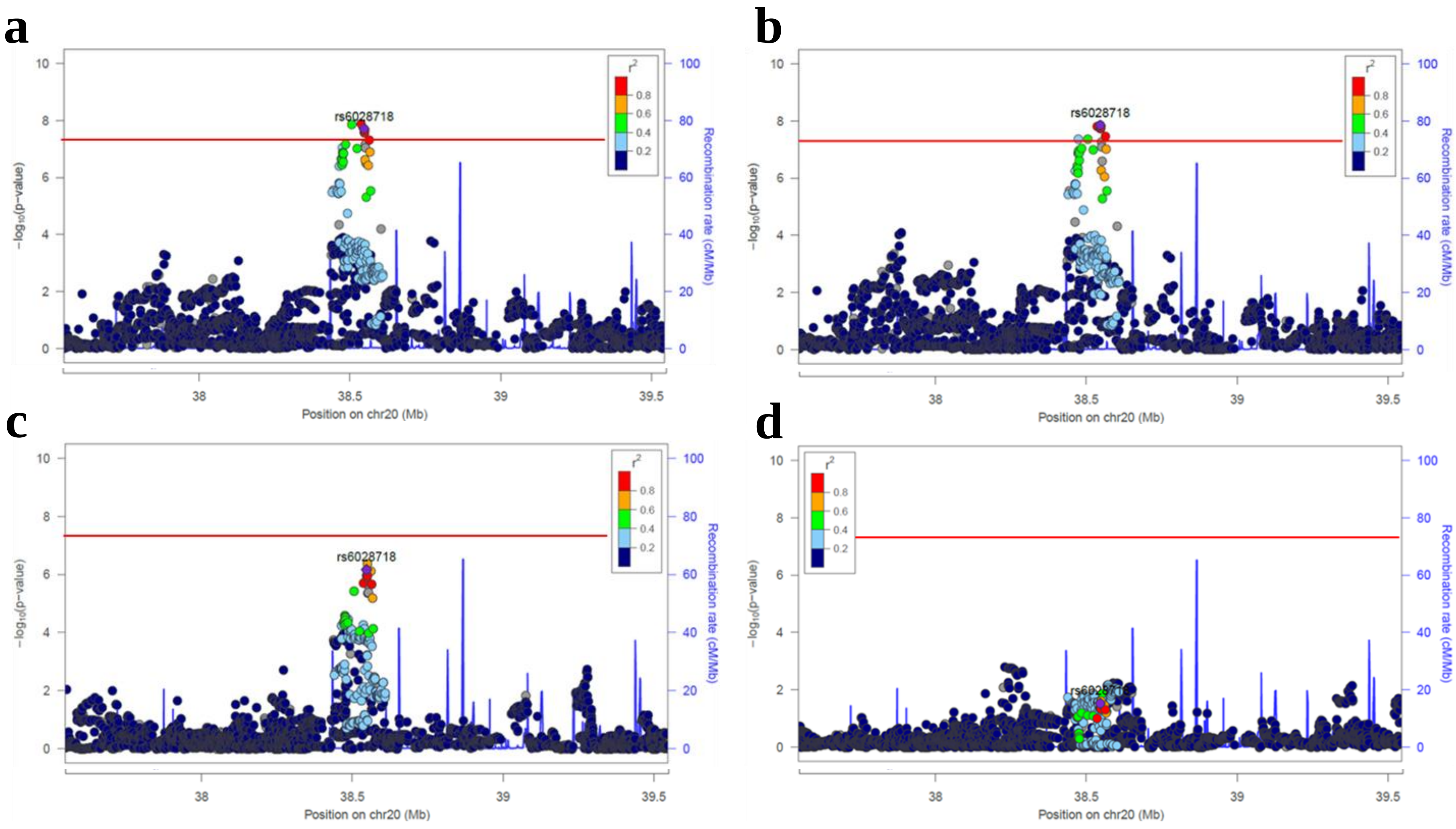

Supplemental Figure 11. Effect size of rs6028718 in each GWAS

Forest plot showing OR and 95% CI of rs6028718 in each GWAS. Box and horizontal line indicate mean OR and 95% CI, respectively. IONFH: idiopathic osteonecrosis of the femoral head, OR: odds ratio, CI: confidence interval. *Controls were matched for alcohol-drinking history (400 ml or more ethanol consumption per week).

Phenotype	OR	95% CI
IONFH	1.32	[1.21, 1.46]
Stratified analysis based on risk factors		
Alcohol-associated	1.56	[1.33, 1.82]
Steroid-associated	1.25	[1.14, 1.36]
Neither-associated	1.31	[1.03, 1.68]
*Stratified analysis based on alcohol intake**		
Alcohol-associated	1.61	[1.36, 1.89]

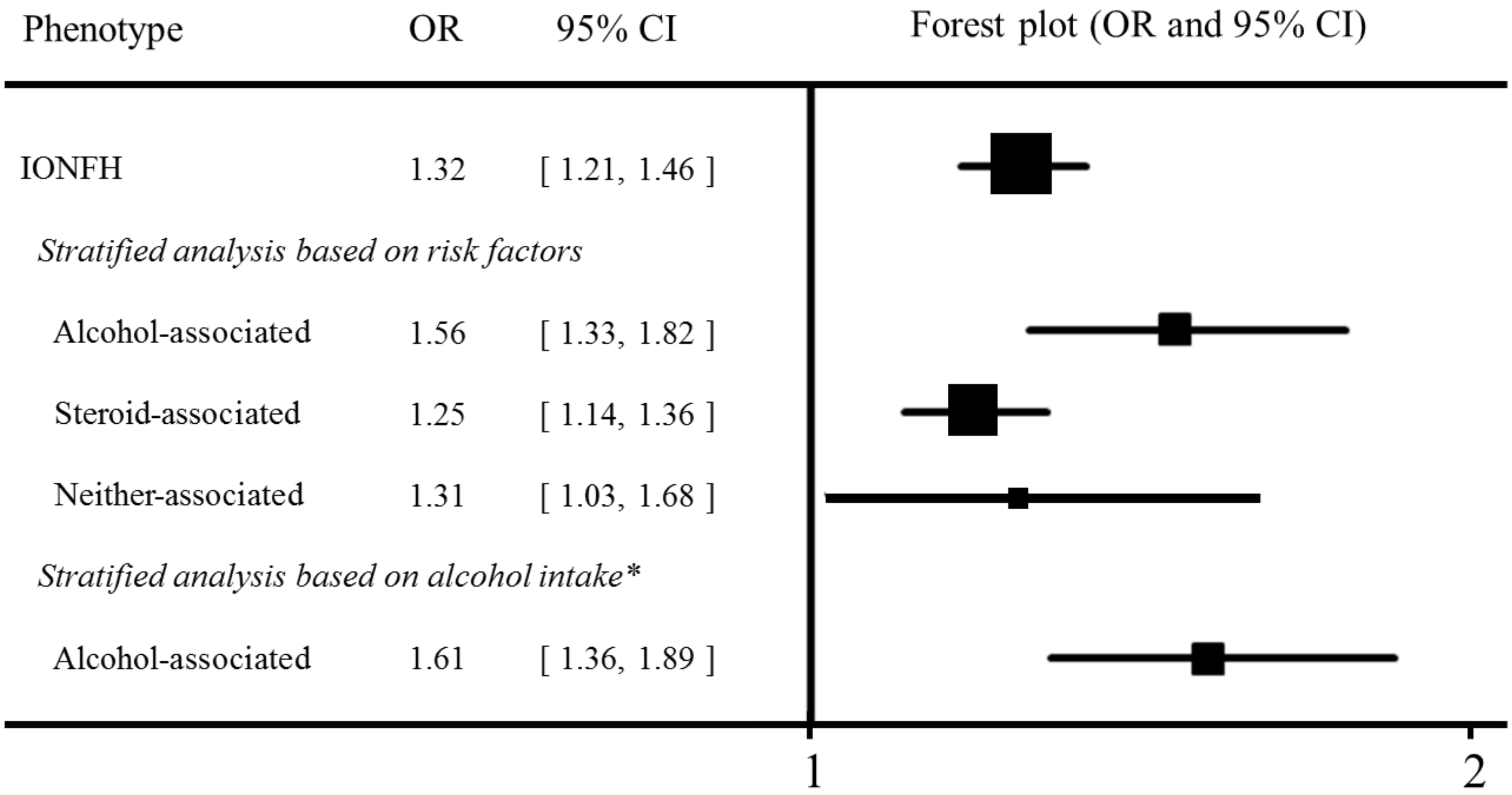

Supplemental Figure 12. Linkage disequilibrium in the 20q12 locus for idiopathic osteonecrosis of the femoral head

The yellow-colored region shows the 20q12 disease-susceptibility locus, containing significantly associated SNPs and surrounded by distinct peaks of recombination rate (> 20 cM/Mb). There were no SNPs in strong linkage disequilibrium with lead GWAS SNP in genic regions around the locus. The red line represented the threshold of genome-wide significance (P = 5 x 10^{-8}). The color intensity reflected the extent of linkage disequilibrium index (r^2) with rs6028718 (in purple). Estimated recombination rates from the hg19/1000 Genomes Project Nov 2014 East Asian reference were shown as light-blue lines.

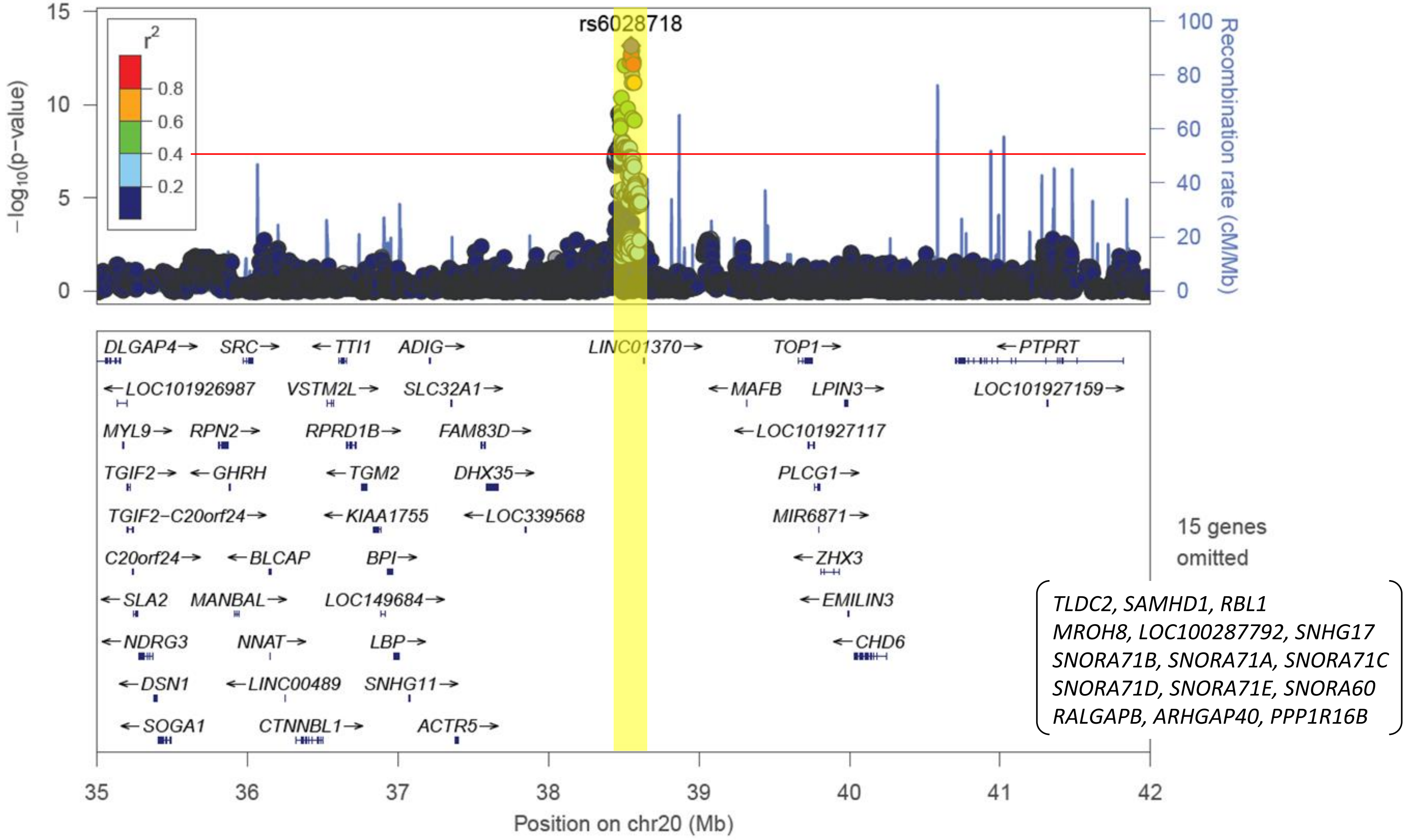

Supplemental Figure 13. Box plots of top 4 eQTL combinations of candidate genes, genome-wide significant SNPs, and tissues available in GTEx

Top 4 eQTL combinations were composed of *LINC01370*, liver, and **(a)** rs62212405 (P_{eQTL} = 0.00018), **(b)** rs2208649 (P_{eQTL} = 0.00024), **(c)** rs2224539 (P_{eQTL} = 0.00049), and **(d)** rs2145943 (P_{eQTL} = 0.0011). Data were obtained from GTEx (accessed 2017 Feb. 21).

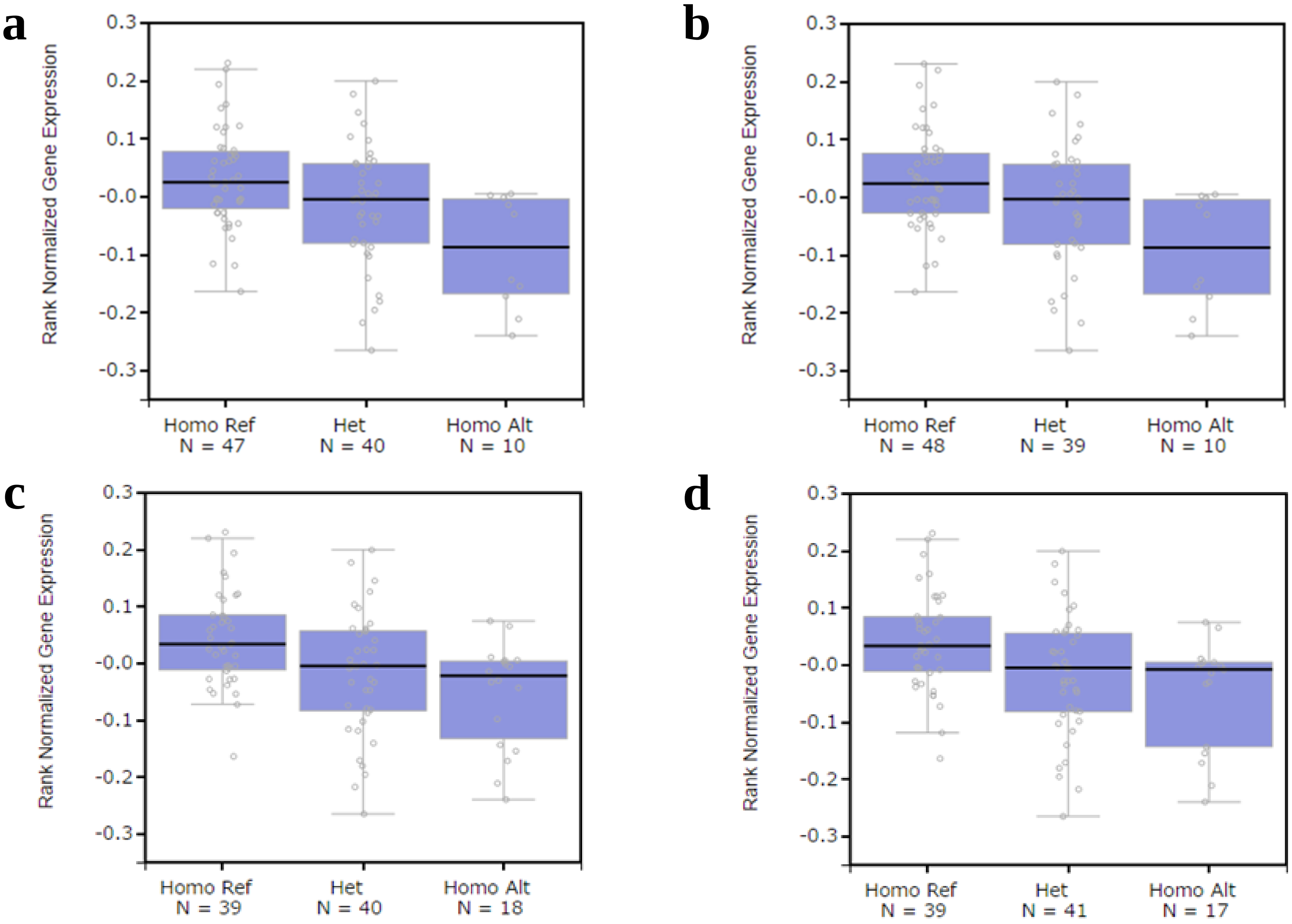

Supplemental Figure 14. LINC01370 expression from public databases

(a) GTEx. LINC01370 (RP11-101E14.2) expression values are shown in RPKM (reads per kilobase of transcript per million mapped reads). **(b)** FANTOM5. Top 10 CAGE expression of LINC01370 (p1@ENST00000432633). Both databases were accessed 2017 Feb. 21.

a

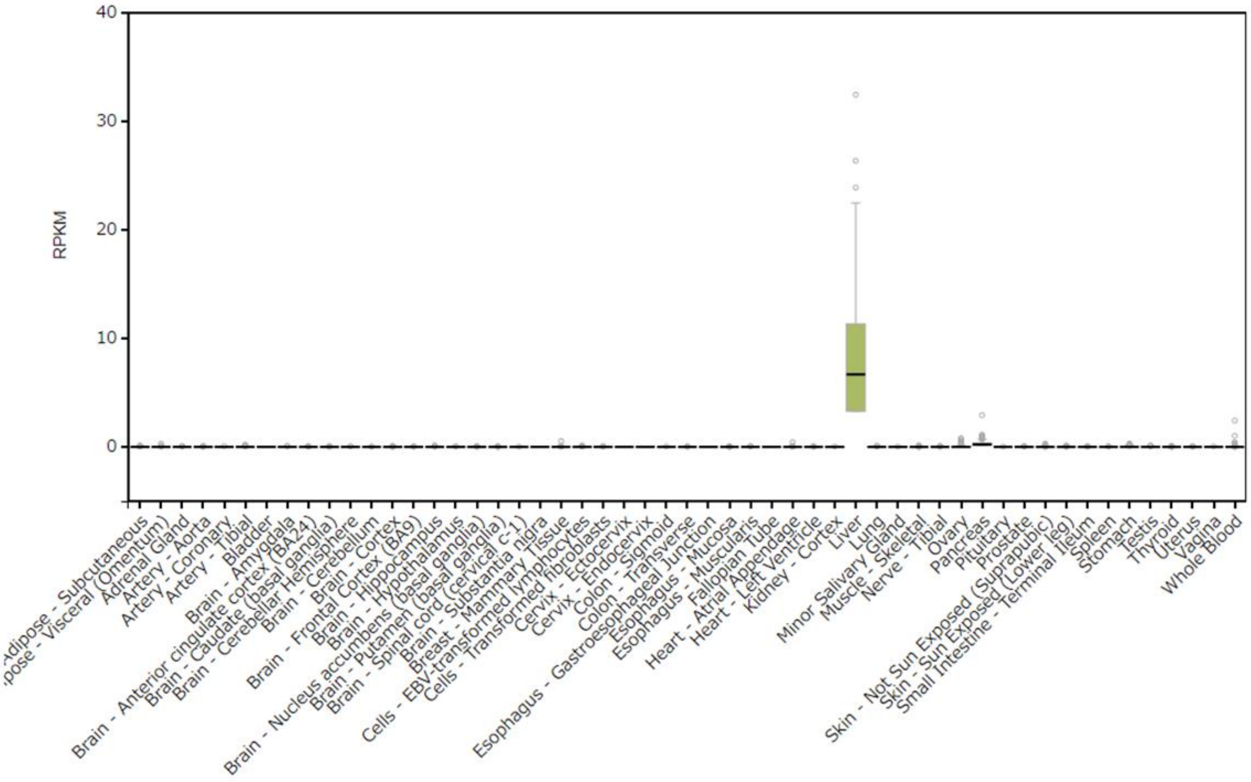

b

Hepatocyte, donor2.CNhs12349.11603-120I1	12.78
liver, adult, pool1.CNhs10624.10018-101C9	9.53
Hepatocyte, donor3.CNhs12626.11684-122I1	9.45
Hepatocyte, donor1.CNhs12340.11523-119I2	8.87
liver, fetal, pool1.CNhs11798.10086-102B5	1.33
stomach, fetal, donor1.CNhs11771.10062-101H8	0.73
SABiosciences XpressRef Human Universal Total RNA, pool1.CNhs10610.10002-101A5	0.62
Universal RNA - Human Normal Tissues Biochain, pool1.CNhs10612.10007-101B4	0.56
keratoacanthoma cell line:HKA-1.CNhs11880.10791-110H8	0.34
ovary, adult, pool1.CNhs10626.10020-101D2	0.08

Supplemental Figure 15. Conditional analysis adjusting for rs671 genotyped data on the 12q24 locus

The regional association plots before **(a, c)** and after **(b, d)** adjusting for rs671 genotyped data on the imputation analysis for idiopathic and alcohol-associated osteonecrosis of the femoral head, respectively. In both analysis, the significant association of the 12q24 locus **(a, c)** disappeared after adjusting **(b, d)**. The red line represented the threshold of genome-wide significance (P = 5 x 10^{-8}). The color intensity reflected the extent of linkage disequilibrium index (r^2) with rs3858704 (in purple). Estimated recombination rates from the hg19/1000 Genomes Project Nov 2014 East Asian reference were shown as light-blue lines.

a

b

c

d

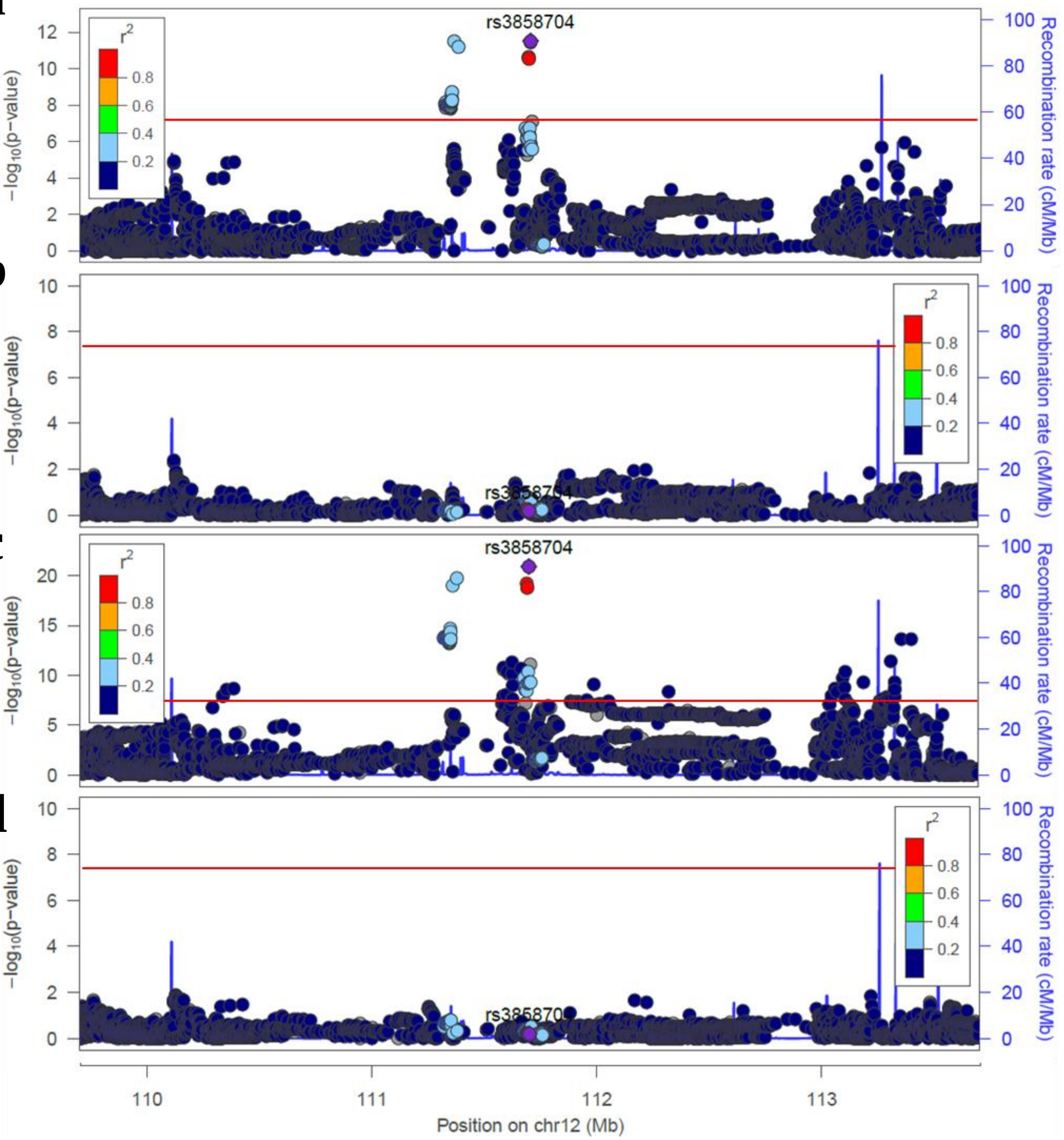

Supplemental Figure 16. Regional association plots of four loci which were previously reported to be associated with steroid-associated osteonecrosis of the femoral head in acute lymphoid leukemia

The association results for steroid-associated osteonecrosis of the femoral head: **(a)** 1q32.3 (target SNP: rs1891059), **(b)** 8q13.1 (rs141059755), **(c)** 9q31.1 (rs10989692), **(d)** 20q13.31 (rs79085477). No loci showed significant associations in our samples. The red line represented the threshold of genome-wide significance ($P = 5 \times 10^{-8}$). Light-blue lines showed estimated recombination rates from the hg19/1000 Genomes Project Nov 2014 East Asian reference. The yellow box represented linkage disequilibrium (LD) block surrounded by distinct peak of recombination rate. The color intensity reflected the extent of LD index (r^2) with the top SNP in LD block or target SNP (in purple). The longitudinal purple line represented the position of target SNP in each locus. There were no genotyped or imputation data about rs1891059 in 1q32.3 **(a)** and rs141059755 in 8q13.1 **(b)**.

a

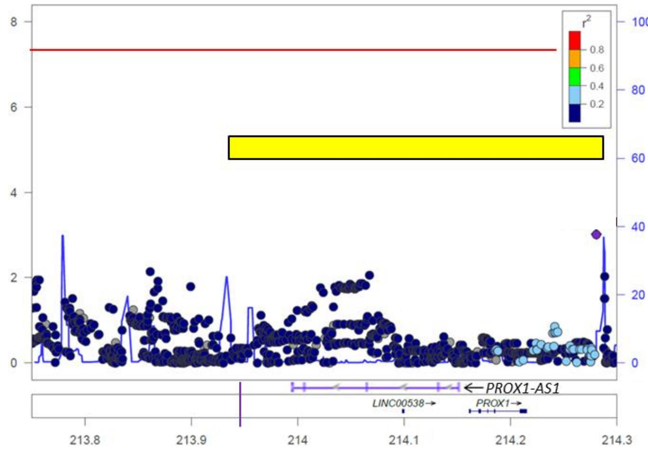

b

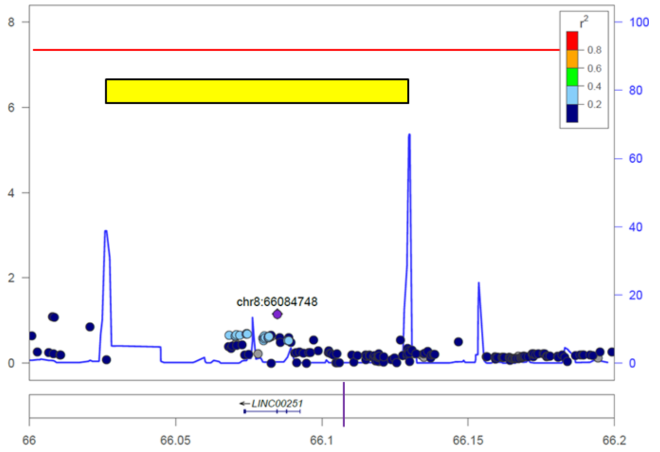

c

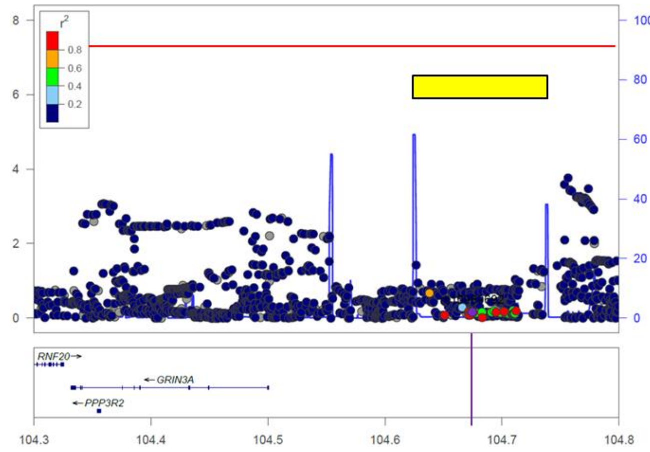

d

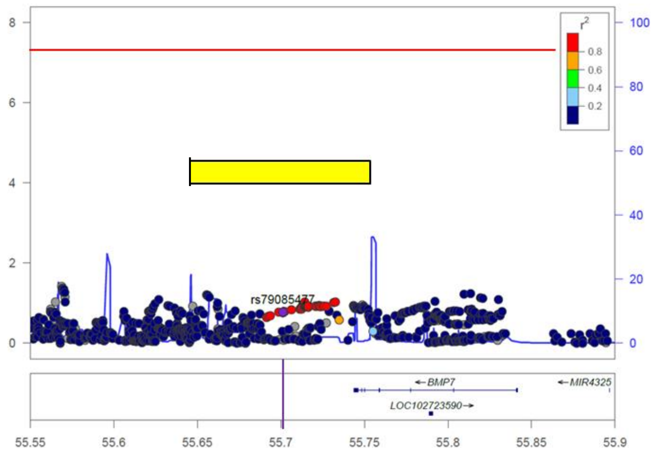

Supplemental Table 1. Sample quality control for GWAS

	IONFH	BBJ	Total
Recruited	1,602	60,000	61,602
Excluded	55	897	952
Call rate < 98%	3	0	3
Close relatedness*	5	889	894
Outlier from East Asia cluster	4	0	4
Gender mismatch	2	8	10
With past history of BMT	41	0	41
Included	1,547	59,103	60,650

*Within third-degree relatedness. IONFH: idiopathic osteonecrosis of the femoral head, BBJ: BioBank Japan, BMT: bone marrow transplantation.

Supplemental Table 2. SNPs with genome-wide significance in the GWAS for idiopathic osteonecrosis of the femoral head

SNP	Chromosome	Cytoband	Position (bp)	Risk allele frequency		*P**	Odds ratio	95% CI	
				Case	Control			Lower	Upper
rs10774610	12	12q24.11	111340243	0.820	0.782	8.71×10^{-9}	1.31	1.20	1.44
rs3782889			111350655	0.822	0.785	1.29×10^{-8}	1.31	1.20	1.44
rs2301610			111353556	0.824	0.786	6.14×10^{-9}	1.32	1.20	1.45
rs4766566		12q24.12	111706877	0.727	0.673	4.53×10^{-12}	1.33	1.23	1.44
rs13042600	20	20q12	38442513	0.611	0.563	4.73×10^{-8}	1.23	1.14	1.32
rs211841			38474802	0.453	0.400	3.91×10^{-10}	1.26	1.17	1.35
rs211840			38474881	0.453	0.400	2.14×10^{-10}	1.26	1.17	1.36
rs211835			38478566	0.501	0.449	4.66×10^{-10}	1.26	1.17	1.35
rs10211813			38487490	0.668	0.609	4.69×10^{-11}	1.29	1.20	1.39
rs11699371			38538542	0.380	0.333	2.38×10^{-8}	1.23	1.15	1.33
rs6065171			38538768	0.567	0.503	4.38×10^{-13}	1.30	1.21	1.40
rs2145943			38563245	0.640	0.574	4.05×10^{-13}	1.32	1.22	1.42
rs6129447			38566077	0.576	0.512	6.83×10^{-13}	1.30	1.21	1.40

**P*-value of genotyped SNPs was calculated by logistic covariate analysis (gender and top 10 principal component scores were used as covariate). CI: confidence interval.

Supplemental Table 3. Top 10 results of eQTL analysis for all combinations of candidate genes, genome-wide significant SNPs and tissues available in GTEx

Gene	SNP	Tissue/Cell	P_{GWAS}	P_{eQTL}	Effect Size
LINC01370	**rs62212405**	**Liver**	**6.30×10^{-10}**	**0.00018**	**-0.45**
LINC01370	**rs2208649**	**Liver**	**4.83×10^{-10}**	**0.00024**	**-0.44**
LINC01370	**rs2224539**	**Liver**	**1.14×10^{-13}**	**0.00049**	**-0.37**
LINC01370	**rs2145943**	**Liver**	**4.27×10^{-13}**	**0.0011**	**-0.36**
MAFB	rs2208649	Lung	4.83×10^{-10}	0.0012	-0.17
DHX35	rs396182	Brain (Hippocampus)	1.97×10^{-8}	0.0012	0.49
MAFB	rs6028703	Transformed fibroblasts	7.63×10^{-13}	0.0018	-0.14
LINC01370	**rs8119989**	**Liver**	**2.88×10^{-8}**	**0.0020**	**-0.34**
LINC01370	**rs12624742**	**Liver**	**2.04×10^{-8}**	**0.0024**	**-0.34**
LINC01370	**rs4812412**	**Liver**	**2.09×10^{-8}**	**0.0024**	**-0.34**

Results related to *LINC01370* were highlighted in bold. P_{GWAS}: *P*-value of GWAS after imputation for idiopathic osteonecrosis of the femoral head. P_{eQTL}: *P*-value of eQTL (expression quantitative trait locus) analysis. eQTL analysis was performed by using GTEx (accessed 2017 Feb. 21).

Supplemental Table 4. Top 10 results of Gene ontology analysis by LncRNA2Function

Gene ontology term name	Frequency (%)		*P*–value	
	Background	Sample	Uncorrected	Corrected
Cellular component				
Extracellular region	2120/20447 (10.4)	131/391 (33.5)	5.84×10^{-36}	2.28×10^{-32}
Extracellular space	841/20447 (4.11)	81/391 (20.7)	2.11×10^{-34}	7.33×10^{-31}
Extracellular region part	1130/20447 (5.53)	82/391 (21.0)	3.18×10^{-26}	4.52×10^{-23}
**High-density lipoprotein particle**	26/20447 (0.13)	18/391 (4.60)	1.08×10^{-25}	1.46×10^{-22}
**Plasma lipoprotein particle**	38/20447 (0.19)	20/391 (5.12)	6.46×10^{-25}	8.40×10^{-22}
**Protein-lipid complex**	39/20447 (0.19)	20/391 (5.12)	1.30×10^{-24}	1.63×10^{-21}
Endoplasmic reticulum part	926/20447 (4.53)	68/391 (17.4)	5.78×10^{-22}	5.15×10^{-19}
Endoplasmic reticulum	1311/20447 (6.41)	77/391 (19.7)	5.04×10^{-19}	3.58×10^{-16}
**Triglyceride-rich lipoprotein particle**	21/20447 (0.10)	13/391 (3.32)	6.65×10^{-18}	3.77×10^{-15}
**Very-low-density lipoprotein particle**	21/20447 (0.10)	13/391 (3.32)	6.65×10^{-18}	3.77×10^{-15}
Biological process				
Single-organism metabolic process	2653/20447 (13.0)	179/391 (45.8)	4.44×10^{-58}	1.38×10^{-53}
Small molecule metabolic process	2193/20447 (10.7)	162/391 (41.4)	7.67×10^{-57}	1.20×10^{-52}
**Lipid metabolic process**	1051/20447 (5.14)	107/391 (27.4)	1.72×10^{-48}	1.79×10^{-44}
Carboxylic acid metabolic process	787/20447 (3.85)	88/391 (22.5)	1.13×10^{-42}	8.84×10^{-39}
Organic acid metabolic process	911/20447 (4.46)	93/391 (23.8)	7.42×10^{-42}	4.64×10^{-38}
**Steroid metabolic process**	244/20447 (1.19)	53/391 (13.6)	1.70×10^{-40}	8.87×10^{-37}
Oxoacid metabolic process	894/20447 (4.37)	89/391 (22.8)	4.33×10^{-39}	1.93×10^{-35}
Xenobiotic metabolic process	149/20447 (0.73)	38/391 (9.72)	5.03×10^{-32}	1.57×10^{-28}
Cellular response to xenobiotic stimulus	150/20447 (0.73)	38/391 (9.72)	6.62×10^{-32}	1.88×10^{-28}
Response to xenobiotic stimulus	152/20447 (0.74)	38/391 (9.72)	1.14×10^{-31}	2.97×10^{-28}

The terms containing ‘lipoprotein’, ‘lipid’ or ‘steroid’ were highlighted in bold. *P*-value of each term was calculated by hypergeometric test and all *P*-values were corrected by the Benjamini-Yekutieli method.

Supplemental Table 5. Top 30 results of pathway analysis by LncRNA2Function

Pathway		Frequency (%)		*P*–value	
Database	Name	Background	Sample	Uncorrected	Corrected
Reactome	Metabolism	1394/20447 (6.82)	129/391 (33.0)	1.18×10^{-54}	1.19×10^{-51}
KEGG	Metabolic pathways	1256/20447 (6.14)	110/391 (28.1)	1.61×10^{-43}	8.14×10^{-41}
KEGG	Retinol metabolism	69/20447 (0.34)	29/391 (7.42)	6.11×10^{-32}	2.06×10^{-29}
KEGG	**Complement and coagulation cascades**	87/20447 (0.43)	30/391 (7.67)	6.68×10^{-30}	1.69×10^{-27}
EHMN	Androgen and estrogen biosynthesis and metabolism	90/20447 (0.44)	30/391 (7.67)	2.18×10^{-29}	4.40×10^{-27}
Reactome	Biological oxidations	151/20447 (0.74)	36/391 (9.21)	2.97×10^{-29}	4.99×10^{-27}
KEGG	**Drug metabolism - cytochrome P450**	76/20447 (0.37)	28/391 (7.16)	6.27×10^{-29}	9.05×10^{-27}
KEGG	**Metabolism of xenobiotics by cytochrome P450**	87/20447 (0.43)	29/391 (7.42)	1.91×10^{-28}	2.41×10^{-26}
KEGG	Chemical carcinogenesis	86/20447 (0.42)	28/391 (7.16)	3.58×10^{-27}	4.02×10^{-25}
EHMN	Tyrosine metabolism	113/20447 (0.55)	30/391 (7.67)	4.63×10^{-26}	4.68×10^{-24}
EHMN	Xenobiotics metabolism	54/20447 (0.26)	23/391 (5.88)	9.90×10^{-26}	9.09×10^{-24}
Reactome	Metabolism of amino acids and derivatives	159/20447 (0.78)	32/391 (8.18)	1.18×10^{-23}	9.97×10^{-22}
Wikipathways	Metapathway biotransformation	190/20447 (0.93)	34/391 (8.70)	2.64×10^{-23}	2.05×10^{-21}
EHMN	Linoleate metabolism	75/20447 (0.37)	24/391 (6.14)	2.99×10^{-23}	2.16×10^{-21}
Wikipathways	**Cytochrome P450**	68/20447 (0.33)	23/391 (5.88)	5.73×10^{-23}	3.86×10^{-21}
Wikipathways	**Complement and coagulation cascades**	60/20447 (0.29)	22/391 (5.63)	6.32×10^{-23}	3.99×10^{-21}
EHMN	Leukotriene metabolism	102/20447 (0.50)	26/391 (6.65)	3.06×10^{-22}	1.82×10^{-20}
Reactome	Phase 1 - Functionalization of compounds	74/20447 (0.36)	23/391 (5.88)	5.31×10^{-22}	2.98×10^{-20}
KEGG	Drug metabolism - other enzymes	53/20447 (0.26)	20/391 (5.12)	2.99×10^{-21}	1.59×10^{-19}
Reactome	Xenobiotics	15/20447 (0.07)	13/391 (3.32)	3.80×10^{-21}	1.92×10^{-19}
EHMN	**C21-steroid hormone biosynthesis and metabolism**	62/20447 (0.30)	21/391 (5.37)	4.31×10^{-21}	2.07×10^{-19}
Reactome	**Cytochrome P450 - arranged by substrate type**	56/20447 (0.27)	20/391 (5.12)	1.10×10^{-20}	5.07×10^{-19}
INOH	Tryptophan degradation	66/20447 (0.32)	21/391 (5.37)	1.94×10^{-20}	8.53×10^{-19}
Reactome	**Metabolism of lipids and lipoproteins**	496/20447 (2.43)	47/391 (12.0)	1.06×10^{-19}	4.46×10^{-18}
Wikipathways	Phase 1 - Functionalization of compounds	55/20447 (0.27)	19/391 (4.86)	2.16×10^{-19}	8.74×10^{-18}
PharmGKB	Phenytoin pathway, Pharmacokinetics	20/20447 (0.10)	13/391 (3.32)	2.58×10^{-18}	1.00×10^{-16}
PID	**Chylomicron-mediated lipid transport**	16/20447 (0.08)	12/391 (3.07)	3.44×10^{-18}	1.24×10^{-16}
PharmGKB	Carbamazepine pathway, Pharmacokinetics	16/20447 (0.08)	12/391 (3.07)	3.44×10^{-18}	1.24×10^{-16}
PID	Xenobiotics	13/20447 (0.06)	11/391 (2.81)	8.20×10^{-18}	2.86×10^{-16}
Wikipathways	**Statin pathway**	33/20447 (0.16)	15/391 (3.84)	9.71×10^{-18}	3.27×10^{-16}

The plausible pathways for idiopathic osteonecrosis of the femoral head were highlighted in bold. *P*-value of each term was calculated by hypergeometric test and all *P*-values were corrected by the Benjamini-Hochberg method.

Supplemental Table 6. Top 10 results of Gene ontology and KEGG pathway analysis by Co-LncRNA

Term and pathway	Number of Genes			Enrichment *P* value		
	WP	CE	Overlap	Nominal	B.H.	Bonf.
Gene ontology (Biological process)						
**Cellular lipid metabolic process**	150	143	7	4.44×10^{-7}	1.97×10^{-5}	2.77×10^{-4}
Translational initiation	119	143	5	1.76×10^{-5}	3.02×10^{-4}	0.011
**Fatty acid beta-oxidation**	36	143	3	2.12×10^{-5}	3.07×10^{-4}	0.013
Viral transcription	82	143	4	3.65×10^{-5}	4.96×10^{-4}	0.022
Translational termination	84	143	4	4.10×10^{-5}	5.13×10^{-4}	0.025
Viral life cycle	93	143	4	6.69×10^{-5}	5.49×10^{-4}	0.041
Translational elongation	93	143	4	6.69×10^{-5}	5.49×10^{-4}	0.041
mRNA metabolic process	225	143	6	7.81×10^{-5}	6.17×10^{-4}	0.048
SRP-dependent co-translational protein targeting to membrane	107	143	4	1.30×10^{-4}	7.59×10^{-4}	0.081
RNA metabolic process	246	143	6	1.35×10^{-4}	7.78×10^{-4}	0.084
KEGG pathway						
**Fatty acid metabolism**	42	143	4	1.31×10^{-6}	1.16×10^{-4}	1.16×10^{-4}
PPAR signaling pathway	69	143	4	1.57×10^{-5}	6.95×10^{-4}	0.001
Ribosome	88	143	4	5.14×10^{-5}	0.001	0.004
Non small cell lung cancer	54	143	3	1.06×10^{-4}	0.001	0.009
Glioma	65	143	3	2.20×10^{-4}	0.002	0.019
MAPK signaling pathway	267	143	6	2.24×10^{-4}	0.002	0.019
Pancreatic cancer	70	143	3	2.93×10^{-4}	0.002	0.025
Melanoma	71	143	3	3.09×10^{-4}	0.002	0.027
Chronic myeloid leukemia	73	143	3	3.44×10^{-4}	0.002	0.030
Peroxisome	78	143	3	4.43×10^{-4}	0.003	0.039

The plausible pathways for idiopathic osteonecrosis of the femoral head were highlighted in bold. WP: genes analyzed within pathway, CE: co-expressed genes with LINC01370, B.H.: corrected by the Benjamini & Hochberg method, Bonf.: corrected by the Bonferroni method.

Supplemental table 7. Biological pathways or gene sets significantly associated with idiopathic osteonecrosis of the femoral head identified by MAGENTA

Database	Pathway or gene set	Analyzed genes	95% cutoff (Top 5%)			
			Nominal *P*-value	FDR	Expected genes	Observed genes
GOTERM	Positive regulation of B cell proliferation	20	2.50×10^{-5}	2.65×10^{-2}	1	7
Ingenuity	Chemokine signaling	21	4.00×10^{-4}	1.45×10^{-2}	1	6
Ingenuity	Fc epsilon RI signaling	17	1.00×10^{-3}	1.63×10^{-2}	1	5
Ingenuity	Neuregulin signaling	25	6.80×10^{-3}	3.86×10^{-2}	1	5
Ingenuity	**VEGF signaling**	16	7.60×10^{-3}	4.48×10^{-2}	1	4
Ingenuity	Cell cycle G1/S checkpoint regulation	17	8.80×10^{-3}	4.19×10^{-2}	1	4
Ingenuity	JAK-STAT signaling	10	1.05×10^{-2}	3.91×10^{-2}	1	3

The plausible pathways for idiopathic osteonecrosis of the femoral head were highlighted in bold. FDR: false discovery rate

Supplemental Table 8. rs671 genotype of the subjects

Subject	Number	Genotype count			Allele frequency		Heterogeneity		
		AA	AG	GG	A	G	Observed	Estimated	P_{HWE}*
IONFH	1,547	67	436	1,041	0.185	0.815	0.282	0.301	0.018
Stratification based on risk factors									
Alcohol-associated	351	0	29	322	0.041	0.959	0.083	0.079	1
Steroid-associated	1,058	62	359	635	0.229	0.771	0.340	0.353	0.256
Neither-associated	132	5	45	81	0.210	0.790	0.344	0.332	0.798
BioBank Japan	59,103	3,900	21,666	33,493	0.250	0.751	0.367	0.375	8.99×10^{-7}
Heavy alcohol drinker[#]	3,647	3	517	3,124	0.072	0.928	0.142	0.133	5.15×10^{-6}

IONFH: idiopathic osteonecrosis of the femoral head, P_{HWE}: P-value of Hardy-Weinberg equilibrium. *The threshold in this study was 1×10^{-6}. [#]400 ml/week or more pure ethanol consumption.

Supplemental Table 9. Association of rs671 in IONFH

	Case	Control	Risk allele frequency		*P*	Odds ratio	95% CI	
			Case	Control			Lower	Upper
IONFH	1,547	59,103	0.815	0.750	6.40×10^{-20}	1.54	1.40	1.69
Stratified analysis based on risk factors								
Alcohol-associated	351	59,103	0.959	0.750	3.26×10^{-28}	8.12	5.59	11.8
Steroid-associated	1,058	59,103	0.771	0.750	0.00304	1.17	1.05	1.30
Neither-associated	132	59,103	0.790	0.750	0.0501	1.35	1.00	1.82
*Stratified analysis based on alcohol intake**								
Alcohol-associated	351	3,647	0.959	0.928	0.00127	1.91	1.29	2.84

IONFH: idiopathic osteonecrosis of the femoral head, CI: confidence interval. *P*-value was calculated by logistic covariate analysis (Gender and top 10 principal components were used as covariate). *Controls were matched for alcohol-drinking history (400 ml or more ethanol consumption per week).

Supplemental Table 10. Association in our GWAS of four SNPs reported in previous GWAS for steroid-associated osteonecrosis of the femoral head in children with acute lymphoblastic leukemia[#]

SNP	Position	Cytoband	Nearest gene	Minor allele frequency in each ethnic group					*P*-value*
				AFR	AMR	EUR	SAS	EAS	
rs1891059	chr1:213,946,009	1q32.3	*PROX1-AS1*	0.026	0.033	0.059	0.016	0	No data
rs141059755	chr8:66,107,605	8q13.1	*LINC00251*	0.001	0.007	0.001	0	0	No data
rs10989692	chr9:104,674,555	9q31.1	*GRIN3A*	0.319	0.071	0.105	0.025	0.022	0.682
rs79085477	chr20:55,701,215	20q13.31	*BMP7*	0.058	0.007	0.008	0.035	0.068	0.170

[#]Blood 2015 (ref. 25) and 2016 (ref. 26) by Karol *et al*.. **P*-value in our GWAS after imputation for steroid-associated osteonecrosis of the femoral head. Allele frequency data were derived from 1000 Genomes Project Phase 3 (AFR: African, AMR: American, EUR: European, SAS: South Asian, EAS: East Asian).
